# Renal Safety of Topical Diclofenac in Adults with Increased Renal Risk: A Systematic Review

**DOI:** 10.3390/medicina62081606

**Published:** 2026-08-21

**Authors:** Eric-Oliviu Coșovanu, Elena-Teona Coșovanu, Teodora Ana Balan, Cezar Ilie Foia, Cosmin Gabriel Tarțău, Tiberiu Lunguleac, Aurelian-Bogdan Stana, Antoneta Dacia Petroaie, Simona Eliza Giușcă, Elena Adorata Coman, Demetra Socolov, Raluca Anca Balan, Ramona Gabriela Ursu, Irina-Draga Căruntu, Liliana Mititelu-Tarțău

**Affiliations:** “Grigore T. Popa” University of Medicine and Pharmacy, 16 Universitatii Street, 700115 Iasi, Romania; cosovanu_eric-oliviu@d.umfiasi.ro (E.-O.C.); balan.teodora-ana@d.umfiasi.ro (T.A.B.); cezar030699@gmail.com (C.I.F.); cosmin.tartau@gmail.com (C.G.T.); tiberiu.lunguleac1@umfiasi.ro (T.L.); aurelian.stana@umfiasi.ro (A.-B.S.); antoneta.petroaie@umfiasi.ro (A.D.P.); simona-eliza.giusca@umfiasi.ro (S.E.G.); elena.coman@umfiasi.ro (E.A.C.); demetra.socolov@umfiasi.ro (D.S.); raluca.balan@umfiasi.ro (R.A.B.); irina.caruntu@umfiasi.ro (I.-D.C.); lylytartau@yahoo.com (L.M.-T.)

**Keywords:** topical diclofenac, non-steroidal anti-inflammatory drugs, acute kidney injury, chronic kidney disease, renal safety, osteoarthritis, pharmacovigilance, drug safety, systematic review, GRADE

## Abstract

*Background and Objectives:* Topical diclofenac is recommended over oral non-steroidal anti-inflammatory drugs (NSAIDs) for osteoarthritis, particularly in older adults and those with comorbidities, on the assumption that low systemic absorption limits renal effects. Whether this presumed safety extends to patients already at increased risk of kidney injury has not been systematically evaluated. This review assessed the evidence on renal outcomes of topical diclofenac in adults. *Materials and Methods:* We conducted a systematic review (PROSPERO CRD420261393454) of studies reporting renal outcomes after topical diclofenac exposure. PubMed, Embase, Web of Science, and Scopus were searched from inception to 19 April 2026. Studies were stratified a priori into increased-renal-risk and general populations and synthesised separately, without pooling. Risk of bias was assessed with RoB 2, ROBINS-I, and JBI tools, certainty with GRADE, and synthesis followed the SWiM framework. *Results:* Eighteen studies, contributing data from more than 500,000 participants, were included; 14 underwent primary risk-of-bias appraisal (three at low, three at serious or high risk). In general populations, topical diclofenac produced little to no change in serum creatinine or creatinine clearance and smaller renal effects than oral diclofenac, providing high-certainty evidence of a favourable profile. In increased-renal-risk populations, one adjusted cohort reported higher acute kidney injury (AKI) risk among topical NSAID users, although exposure was predominantly to non-diclofenac agents; within the same cohort, topical NSAIDs carried lower risk than systemic NSAIDs. No study evaluated early renal injury biomarkers; certainty was moderate owing to indirectness. *Conclusions:* Relative to oral diclofenac, topical diclofenac shows a favourable renal safety profile, with high-certainty evidence in general populations. In adults at increased renal risk, moderate-certainty evidence derived from predominantly non-diclofenac exposure over short observation windows cannot exclude a modest excess of any-stage AKI; renal risk therefore appears reduced rather than absent. Diclofenac-specific studies in chronic kidney disease using sensitive biomarkers and longer follow-up are needed.

## 1. Introduction

### 1.1. Rationale

Acute and chronic musculoskeletal pain are among the most common indications for analgesic therapy in adults, and NSAIDs remain central to their pharmacological management across a broad range of conditions, including osteoarthritis, soft-tissue injury, and regional pain syndromes [1,2,3]. NSAIDs exert their therapeutic effects through inhibition of cyclo-oxygenase (COX) enzymes and subsequent suppression of prostaglandin synthesis. However, this same mechanism underlies the organ toxicity that limits their use, particularly nephrotoxicity [4]. In the kidney, prostaglandins maintain afferent arteriolar vasodilatation and preserve glomerular perfusion during periods of reduced renal blood flow. Their inhibition may therefore precipitate a haemodynamically mediated decline in glomerular filtration, potentially accompanied by tubular injury and disturbances in sodium and potassium homeostasis [4]. Epidemiological studies have consistently associated systemic NSAID exposure with AKI, acute renal failure, and related hospitalisations, with the greatest absolute risk observed among older adults, patients with CKD, and those receiving interacting cardiovascular medications [5,6,7,8]. Consequently, renal tolerability is a major determinant of NSAID safety in these populations.

Diclofenac is among the most widely used NSAIDs worldwide and occupies a distinctive position within the class. As a phenylacetic acid derivative with relative COX-2 selectivity, it combines potent anti-inflammatory activity with well-recognised gastrointestinal, cardiovascular, and renal adverse effects [9,10]. Following oral administration, diclofenac is rapidly absorbed, extensively protein-bound, and undergoes substantial hepatic metabolism, while therapeutic concentrations persist in synovial tissues longer than in plasma, contributing to sustained clinical efficacy despite a short plasma half-life [9,11]. Although additional pharmacological actions have been described, inhibition of prostaglandin synthesis remains the principal mechanism responsible for both its therapeutic effects and its impact on renal autoregulation [9,10].

The distinction between oral and topical administration is therefore clinically important. Oral, intramuscular, and intravenous administration expose the kidney to systemic concentrations capable of interfering with prostaglandin-dependent renal haemodynamics. In contrast, topical diclofenac is designed to concentrate within local tissues while limiting systemic exposure [12,13]. Following application to intact skin, the drug penetrates the stratum corneum and accumulates in underlying tissues, reaching synovial structures at concentrations sufficient to support local efficacy [14,15]. Pharmacokinetic studies show that only a few per cent of the administered dose reaches the systemic circulation, so plasma concentrations after topical administration are substantially lower than after oral therapy [9,16,17,18]. This reduction in systemic exposure provides the pharmacological rationale for improved systemic tolerability, including potentially lower renal toxicity [19]. Because topical and oral formulations contain the same active molecule, comparisons between these routes offer a useful framework for evaluating the contribution of systemic exposure to renal risk.

This pharmacological profile underpins the prominent role of topical NSAIDs in contemporary osteoarthritis management. Both OARSI and American College of Rheumatology guidelines recommend topical NSAIDs as first-line therapy for knee osteoarthritis and favour their use when minimising systemic exposure is desirable [20,21]. Similar recommendations appear in national and population-specific guidance, particularly for older adults [22,23]. Accordingly, topical diclofenac is widely regarded as a safer alternative to oral NSAID therapy. This perception is supported by clinical trials and observational studies reporting favourable overall tolerability [24,25]. However, these studies were designed primarily to evaluate efficacy and general safety rather than renal outcomes, and renal endpoints have rarely been reported in patients with pre-existing risk factors for kidney injury [26,27].

### 1.2. Knowledge Gap

Despite substantial mechanistic and regulatory attention to renal effects, the evidence base for topical diclofenac has been developed primarily around analgesic efficacy and overall tolerability rather than renal safety in the patients most susceptible to renal injury. Cochrane reviews of topical NSAIDs for acute and chronic musculoskeletal pain have demonstrated analgesic efficacy broadly comparable to oral therapy, but renal events were evaluated only within aggregate adverse event profiles and not as dedicated safety outcomes [28,29,30]. Meta-analyses focused specifically on topical diclofenac reached similar conclusions regarding efficacy, while reporting renal outcomes only in generic safety summaries and without stratification according to baseline renal risk [31,32,33]. Likewise, the most comprehensive safety synthesis of topical NSAIDs in osteoarthritis catalogued renal events using standardised adverse event terminology as part of an overall safety assessment, and a pooled safety analysis restricted to topical diclofenac did not distinguish participants at increased risk of renal injury [34,35]. A broader systematic review of topical NSAID safety reported similar limitations [36].

These limitations largely reflect the characteristics of the underlying evidence base. The randomised trials that underpin efficacy estimates and clinical recommendations generally excluded patients with clinically significant renal impairment, resulting in limited representation of the population most vulnerable to NSAID-associated alterations in renal haemodynamics. In contrast, the most detailed renal data available for topical diclofenac originate from populations with normal kidney function. Pharmacokinetic studies have quantified systemic exposure and renal handling directly, but almost exclusively in healthy volunteers [16,18]. Similarly, perioperative studies of systemic diclofenac have measured glomerular filtration and renal blood flow under controlled conditions, predominantly in patients without pre-existing renal disease [37,38]. Consequently, the available evidence is asymmetric: renal outcomes have been measured most rigorously in populations at low baseline renal risk, whereas populations at increased risk remain poorly characterised.

The clinical relevance of this gap is supported by several observations. In individuals with preserved renal function, short perioperative courses of systemic diclofenac have generally produced little or no measurable change in glomerular filtration rate or renal blood flow [37,39]. Similar findings were reported in a controlled study evaluating diclofenac and postoperative prostacyclin generation [38]. These observations suggest that clinically relevant nephrotoxicity may depend on underlying physiological vulnerability rather than representing a uniform drug effect. However, evidence from higher-risk settings remains limited. Case reports have described AKI and acute renal failure following topical NSAID use, including in patients with recognised predisposing factors, indicating that topical administration may reduce systemic exposure without eliminating renal risk [40,41]. Clinical reviews of topical NSAID toxicity have reached similar conclusions [42]. Regulatory labelling also cautions against use in CKD, hypovolaemia, and concurrent renin–angiotensin system blockade, while observational studies have demonstrated substantially increased risks of AKI when NSAIDs are combined with a renin–angiotensin system (RAS) inhibitor and a diuretic, the so-called “triple-whammy” interaction [43,44].

Diclofenac is a particularly relevant candidate for focused evaluation because it combines relatively high cutaneous bioavailability with widespread clinical use. Nevertheless, in the absence of a synthesis specifically addressing renal-risk populations, current recommendations regarding analgesic use in CKD continue to rely largely on indirect evidence and precautionary guidance [45]. To our knowledge, no PRISMA-compliant systematic review has evaluated renal safety as the primary outcome in adults at increased risk of renal injury while integrating randomised and observational evidence within a single eligibility framework. The present review was designed to address this gap.

### 1.3. Objectives

The primary objective of this systematic review was to synthesise the available evidence on the renal safety profile of topical diclofenac in adults at increased risk of renal injury. A secondary objective was to characterise the renal safety profile of topical diclofenac in general adult and healthy-volunteer populations and to report these findings as a separate comparative reference stratum.

This review therefore addressed a single question: In adults at increased renal risk, what is the renal safety profile of topical diclofenac compared with oral diclofenac and other relevant comparators? Particular emphasis was placed on serum creatinine and other measures of renal function.

### 1.4. PECO Framework

The review question was operationalised using a PECO framework, applied in full in Section 2.2. The population comprised adults aged ≥18 years with at least one condition associated with increased renal risk: CKD defined by KDIGO criteria, reduced baseline estimated glomerular filtration rate from a validated creatinine-based equation, age ≥65 years, diabetes mellitus or prediabetes, heart failure, hypovolaemia, nephrotoxic co-medication or polypharmacy, cirrhosis, “triple-whammy” exposure, or pharmacologically treated arterial hypertension [46,47,48]. The exposure was topical diclofenac applied to intact skin for a local musculoskeletal indication, in any approved formulation, salt form, dose, duration, or application area; eligible studies required either at least 7 days of exposure or a documented renal assessment after a single application. Oral diclofenac was the principal comparator, with other systemic diclofenac formulations, placebo, vehicle, and no treatment as secondary comparators, and other topical agents as tertiary comparators contributing within-arm data only. Renal outcomes were organised into four predefined tiers, from a primary renal outcome to a formal evidence gap tier.

## 2. Materials and Methods

### 2.1. Study Design and Registration

This systematic review was conducted between March and June 2026 and is reported in accordance with the PRISMA 2020 checklist [49]. The review protocol was registered in PROSPERO (CRD420261393454) prior to data extraction. The protocol is publicly accessible through the PROSPERO registry under the same registration number. No amendments were made to the registered protocol during the conduct of the review.

The synthesis framework and the rationale for not conducting a meta-analysis are given in Section 2.5. Four bibliographic databases were searched from inception to 19 April 2026; sources and full strategies are described in Section 2.3 and Section 2.4.

### 2.2. Eligibility Criteria

Eligibility was defined according to a PECO framework. The population comprised adults aged ≥18 years receiving topical diclofenac. The exposure was topical diclofenac applied to intact skin. Comparators included systemic diclofenac, placebo or vehicle, no active treatment, or other topical agents (for within-arm analyses only). The outcome was renal safety. Eligible studies were assigned to one of two predefined strata: a renal-risk stratum (Stratum A) and a general or healthy-volunteer stratum (Stratum B). At least one renal outcome was required for inclusion in either stratum.

#### 2.2.1. Inclusion Criteria

Studies were eligible for the renal-risk stratum if they included adults meeting at least one predefined renal-risk condition. These criteria were grouped into 10 categories.

CKD was defined according to KDIGO 2024 criteria and included sustained eGFR < 60 mL/min/1.73 m^2^ for ≥3 months, persistent albuminuria, structural or histological renal abnormalities, renal transplantation, and persistent urinary, tubular, or electrolyte abnormalities [46].

Additional renal-risk criteria included: (i) reduced baseline eGFR <60 mL/min/1.73 m^2^; (ii) age ≥65 years; (iii) diabetes mellitus, prediabetes, impaired fasting glucose, or impaired glucose tolerance (ADA/WHO definitions); (iv) heart failure of any NYHA class; (v) hypovolaemia or volume depletion; (vi) concomitant exposure to nephrotoxic medications or clinically relevant polypharmacy (including renin–angiotensin system inhibitors, diuretics, other NSAIDs, lithium, calcineurin inhibitors, aminoglycosides, iodinated contrast, foscarnet, and use of ≥3 haemodynamically active medications); (vii) recent AKI within 12 months with recovery; (viii) exposure to the “triple-whammy” combination, analysed as a prespecified subgroup; and (ix) pharmacologically treated arterial hypertension.

Two further conditions (untreated hypertension and decompensated cirrhosis or nephrotic syndrome) were included solely in sensitivity analyses and were not applied as primary eligibility criteria. Studies enrolling general adult or healthy-volunteer populations without renal-risk enrichment were eligible for the comparative reference stratum if at least one renal function parameter was reported.

#### 2.2.2. Exclusion Criteria

Studies were excluded if they enrolled paediatric populations (<18 years), patients with end-stage kidney disease (CKD G5; eGFR <15 mL/min/1.73 m^2^) or receiving chronic dialysis (G5D), or patients with active AKI at baseline.

Active AKI was defined as KDIGO stage ≥1 within the preceding 7 days, ongoing septic shock or vasopressor support, ongoing microbiologically confirmed sepsis, or sustained oliguria (<0.5 mL/kg/h for ≥6 h).

Studies were also excluded if eligible subgroups could not be disaggregated when subgroup analysis was required.

Intervention-related exclusions included ophthalmic or otic diclofenac, transmucosal, rectal, intra-articular, or device-assisted delivery (e.g., iontophoresis, phonophoresis, microneedles, electroporation, laser-assisted delivery), as well as combination products without separate diclofenac reporting.

Design-related exclusions included preclinical studies, case reports and case series with <5 participants, conference abstracts without full text, editorials, letters, commentaries, and narrative or systematic reviews (retained only for reference screening).

### 2.3. Information Sources

Four bibliographic databases were searched: MEDLINE (via PubMed), Embase (via Embase.com), Web of Science Core Collection, and Scopus. The Cochrane Central Register of Controlled Trials (CENTRAL) was not searched directly due to institutional access limitations. This was partially mitigated by overlap across the selected databases and by consultation of trial registries; implications are discussed in the Section 4.6. In addition, ClinicalTrials.gov and the WHO International Clinical Trials Registry Platform (ICTRP) were searched for ongoing or unpublished studies. Pharmacovigilance signals were explored using the FDA Adverse Event Reporting System (FAERS) and EMA EudraVigilance public dashboards.

Backward and forward citation chasing was performed for all included studies, along with reference screening of relevant systematic reviews identified during the search process.

### 2.4. Search Strategy

The search strategy combined controlled vocabulary (MeSH/Emtree) with free-text terms in the title and abstract fields. The terms were structured into three conceptual domains: diclofenac, topical administration, and renal outcomes. These domains were combined using the Boolean operator AND. Non-human studies were excluded in Embase. Full database-specific search strategies, including retrieval counts at each stage, are provided in Supplementary Material S1. Strategies were internally checked against the PRESS 2015 guideline to ensure consistency of controlled vocabulary mapping, Boolean logic, and syntax across databases. External PRESS peer review by an information specialist was not feasible and is acknowledged as a limitation.

### 2.5. Study Selection, Extraction and Synthesis of Data

All records were analysed after de-duplication. Two reviewers independently screened the titles, abstracts, and full texts. Disagreements were resolved by discussion or by adjudication from a third reviewer when necessary. Reasons for full-text exclusion were recorded in detail. No automated screening, text-mining, or data-extraction tools were used at any stage.

The data were extracted independently by two reviewers using a piloted standardised extraction form. Discrepancies were resolved by consensus. Where required, study authors were contacted for clarification (up to two contact attempts at 2-week intervals). For continuous outcomes, effect estimates were based on mean change from baseline with corresponding measures of dispersion. For dichotomous outcomes, risk ratios or odds ratios with 95% confidence intervals were extracted as reported. No statistical transformation for pooling was performed.

Synthesis followed the Synthesis Without Meta-Analysis (SWiM) framework [50]. A meta-analysis was not conducted due to expected clinical heterogeneity, variability in baseline renal function, and low event rates across studies. Evidence synthesis included structured summary tables of study characteristics, risk of bias, and renal outcomes, as well as direction-of-effect synthesis for studies reporting comparable outcomes in at least three datasets.

The decision not to pool was taken at protocol stage and was reconsidered against the extracted dataset. Continuous serum creatinine and creatinine clearance under head-to-head comparison with oral diclofenac were reported by only two independent trials [51,52], which is below the minimum of three datasets prespecified for even a non-quantitative direction-of-effect synthesis. Those two trials are also the source populations of an already published pooled analysis [53], so combining them here would have reproduced an existing estimate while breaching the de-duplication rule applied across overlapping reports (Section 3.7). The trials further differ in design and comparator structure, one being a double-dummy equivalence trial against oral diclofenac and the other a five-arm trial additionally incorporating placebo, vehicle and combination arms. Finally, no study in the renal-risk stratum reported continuous renal function data, so pooling would have produced a precise summary estimate for the comparative reference stratum alone, leaving the primary review question without one and introducing an asymmetry in apparent precision between strata. Because the direction and magnitude of effect were concordant across both trials and the certainty rating for this outcome was already high, pooling would have refined precision without altering the inference.

A non-pooled forest plot was used to visualise individual study effects for the primary outcome. Results were stratified into two predefined populations: (i) renal-risk populations (primary synthesis) and (ii) general or healthy-volunteer populations (comparative reference), which were analysed separately and not combined. Concordance between strata was interpreted as consistent drug effects across populations, whereas divergence was interpreted as potential effect modification by baseline renal risk.

### 2.6. Risk-of-Bias and Certainty Assessment

Risk of bias was assessed independently by two reviewers, with disagreements resolved by discussion or adjudication by a third reviewer when necessary. Outcome-specific risk of bias was prioritised, with the primary focus on renal outcomes.

Randomised controlled trials were assessed using the revised Cochrane Risk of Bias tool (RoB 2) across five domains: randomisation process, deviations from intended interventions, missing outcome data, measurement of the outcome, and selection of the reported result [54]. The crossover trial was assessed using the corresponding RoB 2 extension, including evaluation of potential carryover effects.

Non-randomised studies of interventions were assessed using ROBINS-I across seven domains: confounding, selection of participants, classification of interventions, deviations from intended interventions, missing data, measurement of outcomes, and selection of the reported result [55]. Single-arm studies were assessed using the Joanna Briggs Institute (JBI) Critical Appraisal Checklist for Case Series [56], as ROBINS-I is not applicable in the absence of a comparator. The Newcastle–Ottawa Scale was not used, in line with current Cochrane guidance.

Each study received an outcome-specific risk-of-bias judgement for the renal endpoint, reflecting the adequacy of pre-specification, measurement, and completeness of renal outcome reporting. A study-level judgement was also recorded for contextual interpretation, primarily reflecting overall methodological conduct. For synthesis purposes, the renal outcome judgement was considered the primary assessment domain and was used in sensitivity analyses and certainty-of-evidence appraisal. In visual representations, the “overall” judgement reflects the renal outcome-specific assessment. For non-randomised and single-arm studies, the renal and study-level judgements were identical, as renal outcomes constituted the primary endpoint of interest. Domain-level and overall judgements were visualised as traffic-light plots using the robvis tool [57].

Post-hoc pooled analyses were not assessed separately for risk of bias, as they relied entirely on data derived from included primary studies. Risk of bias was therefore attributed to the original studies, and duplicate representation of participants was avoided in the synthesis. Studies without reported renal outcomes were excluded at full-text screening and were not subjected to risk-of-bias assessment.

A prespecified sensitivity analysis restricted synthesis to studies judged at low or moderate risk of bias for renal outcomes, excluding studies rated as serious or high risk, in order to evaluate robustness of findings. The results of this sensitivity analysis are reported in Section 3 and interpreted in Section 4.

Risk of bias due to missing evidence was assessed narratively with the ROB-ME framework [58], applied to each synthesised outcome rather than to individual studies. For every renal outcome, the reviewers recorded whether renal parameters had been measured but not reported numerically, and judged whether the omission plausibly depended on the results obtained. Funnel-plot asymmetry and regression-based tests were not applicable, since no outcome was informed by the 10 or more studies these methods require, and no meta-analysis was performed.

Certainty in the body of evidence was rated with the GRADE approach [59], independently by two reviewers, separately for each prespecified population stratum and each main renal outcome; disagreements were resolved by discussion. Because non-randomised studies were appraised with ROBINS-I, all bodies of evidence began at high certainty and were then rated down for risk of bias, inconsistency, indirectness, imprecision, or missing evidence, in accordance with GRADE guidance for ROBINS-I-based assessments [60]. Ratings and their justification are recorded in a Summary of Findings table, with wording following GRADE informative-statement conventions [61].

## 3. Results

### 3.1. Study Selection

**Figure 1:**
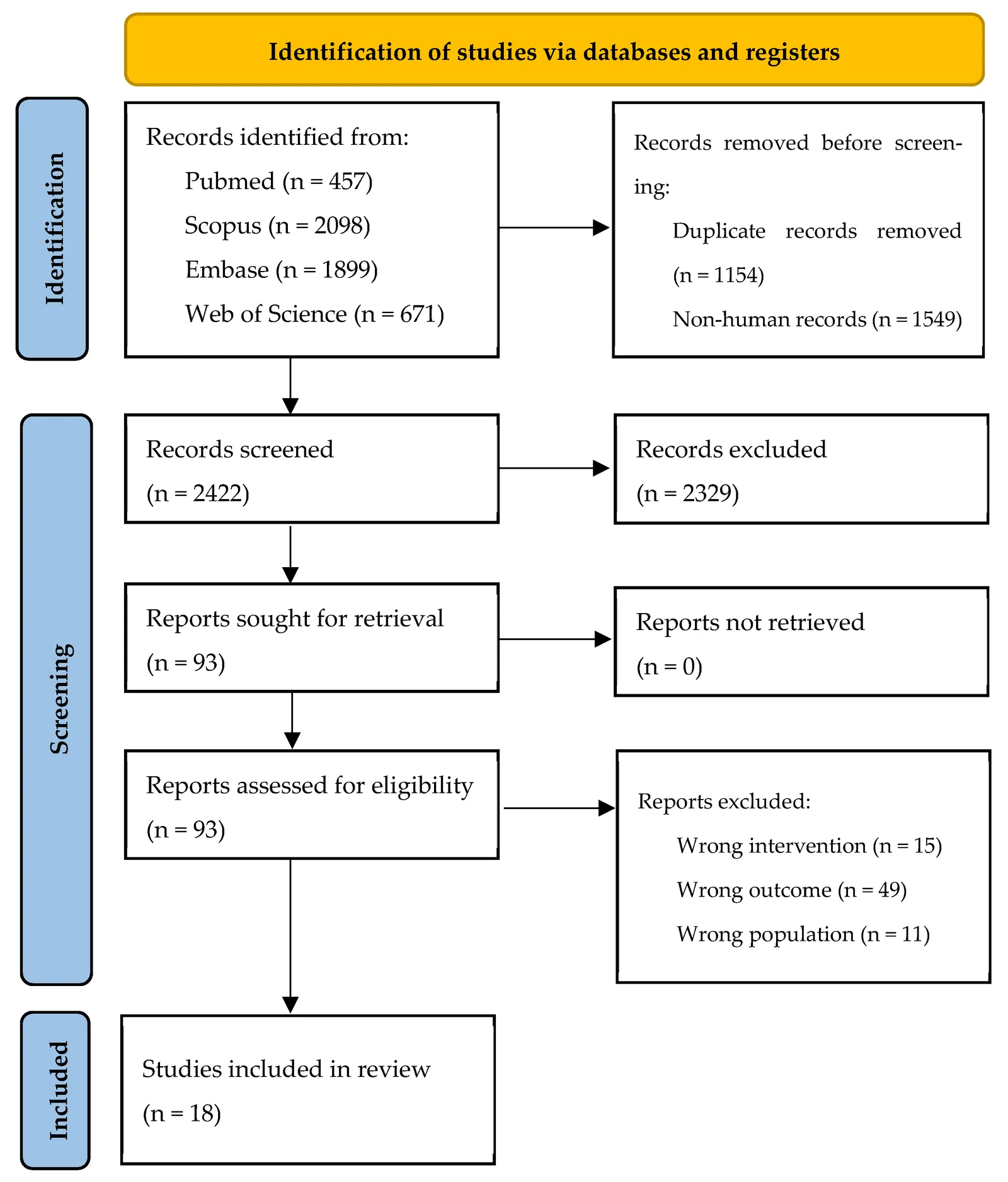
PRISMA 2020 flow diagram for the systematic review. Records were identified from PubMed/MEDLINE (*n* = 457), Scopus (*n* = 2098), Embase (*n* = 1899), and Web of Science (*n* = 671); total *n* = 5125. After removal of duplicate (*n* = 1154) and non-human (*n* = 1549) records, 2422 records were screened and 2329 excluded. Ninety-three reports were sought and assessed for eligibility; 75 were excluded (wrong intervention, *n* = 15; wrong outcome, *n* = 49; wrong population, *n* = 11). A total of 18 studies were included in the review.

The database search identified 5125 records: 457 from PubMed/MEDLINE, 2098 from Scopus, 1899 from Embase, and 671 from Web of Science. After removal of 1154 duplicates and 1549 non-human records, 2422 records remained for title and abstract screening. Of these, 2329 records were excluded at the screening stage, and 93 full-text reports were assessed for eligibility. At full-text assessment, 75 reports were excluded: 15 for inappropriate intervention, 49 for inappropriate outcome, and 11 for inappropriate population. Eighteen studies met the eligibility criteria and were included in the synthesis. The study selection process is presented in the PRISMA 2020 flow diagram (Figure 1).

### 3.2. Risk of Bias of Included Studies

The risk of bias for the renal outcome was the primary appraisal dimension; the assessment procedure is described in Section 2.6. Fourteen studies underwent primary appraisal: nine randomised trials, three non-randomised studies, and two single-arm series. Three were at low risk of bias for the renal outcome, eight at some concerns or moderate risk, and three at serious or high risk. Table 1 lists the studies in each category. The four combined analyses inherited the risk of their source trials and were not counted independently.

The shortfall was concentrated in the measurement and reporting of renal endpoints. A few methodologically sound trials reached low risk of bias for the renal outcome, because renal parameters were both measured and reported [62,63,64]. Others, which were sound for their efficacy outcomes, fell into the some-concerns or serious risk groups for the renal outcome, because creatinine was either not measured or collected but not reported. The two cohort studies were at serious risk, driven by confounding and, for the diclofenac question, by indirect exposure.

This pattern also bears on the risk of bias due to missing evidence, assessed narratively with the ROB-ME framework. In four trials, renal laboratory parameters were collected as part of routine safety monitoring but were reported only qualitatively, as the absence of clinically significant change, without numerical values [62,63,64,70]. Because these unreported data were measured but not made available, and because their omission plausibly depended on their showing no signal, the synthesis is at risk of selective non-reporting of renal results across the evidence base. This missing-evidence bias operates in the direction of reassurance and cannot be quantified from the published record; it is therefore carried forward as a qualifier on every renal safety inference in this review.

The single-arm studies were appraised with the JBI case series checklist, the design-appropriate instrument when no comparator is present (Table 2). For both studies, the renal laboratory data were collected but not reported, with approximately 50% attrition.

Four post-hoc pooled analyses re-evaluated participants already represented in their source trials. They were not appraised independently; their renal risk of bias was inherited from the source trials, and only one representation of each participant set was carried into the synthesis (Table 3).

To prevent double-counting, a single representation of each shared participant set was carried forward. For the diclofenac-versus-oral question the two source trials (Tugwell et al. 2004 [51] and Simon et al. 2009 [52]) were used in place of their pool [53]; for the diclofenac-versus-placebo question, one solution pool was retained [75]; the elderly-subset pool [74] represented the renal-risk reading of the same trials; and Altman et al. 2009 [62] represented the gel programme instead of its pool [76]. The clustering of overlapping reports is detailed in Section 3.7.

In the sensitivity analysis restricted to studies at lower renal risk of bias, the three low-risk trials and the eight some-concerns or moderate studies were retained, but the entire Level 1–2 renal-risk-population evidence (Teo et al. 2023 [72] and Sicignano et al. 2025 [73]) was removed, since both cohorts were at serious risk. The reassuring general population signal therefore persisted in the lower-risk subset, whereas the renal-risk stratum had no lower-risk evidence to fall back on. This trade-off is considered in Section 4.

**Figure 2:**
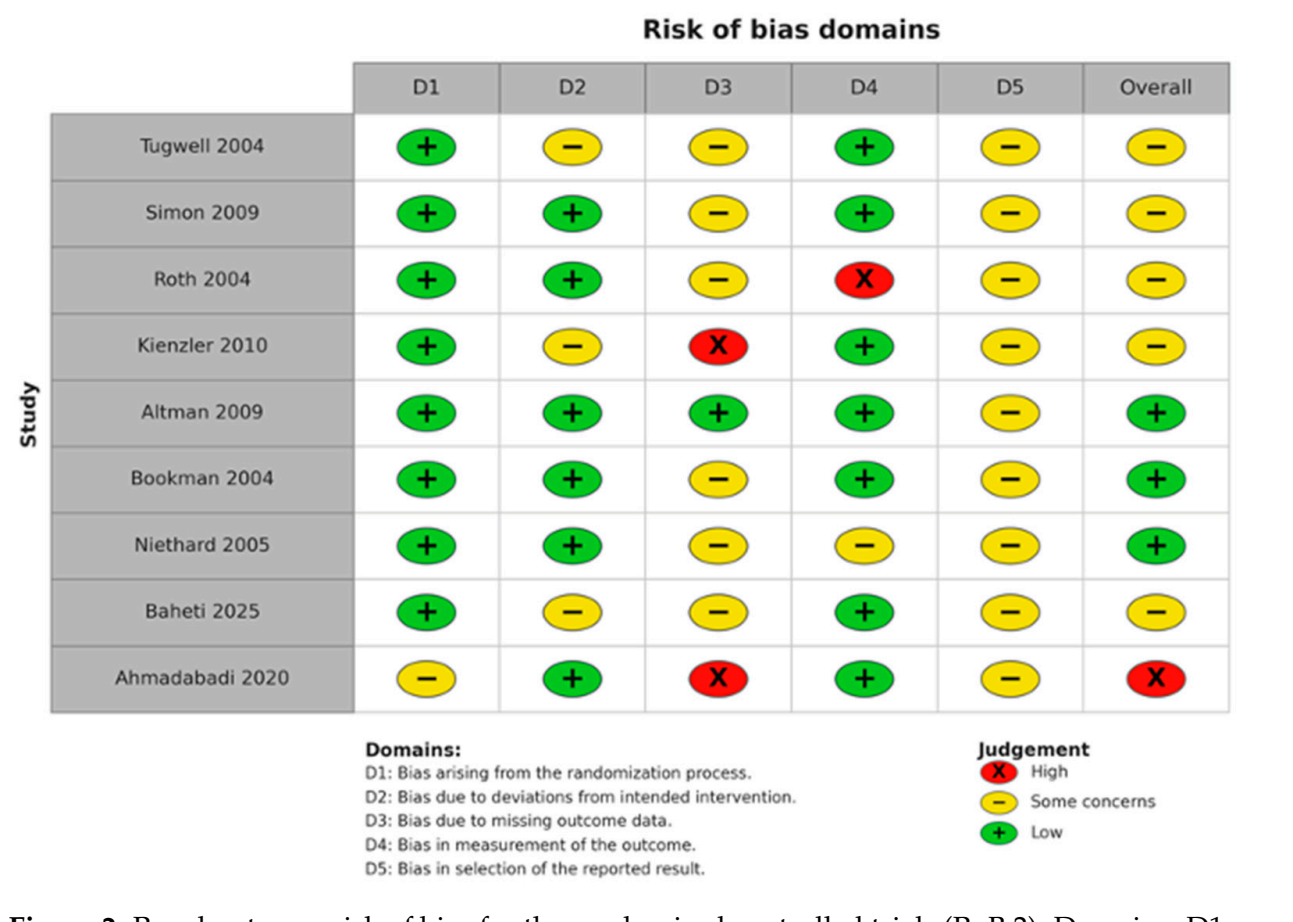
Renal outcome risk of bias for the randomised controlled trials (RoB 2). Domains: D1, randomisation; D2, deviations from intended interventions; D3, missing outcome data; D4, measurement of the renal outcome; D5, selection of the reported result. The Overall column shows the renal outcome rating. Kienzler et al. 2010 [66] was assessed with the crossover variant. Green, low; yellow, some concerns; red, high [51,52,62,63,64,65,67,71].

**Figure 3:**
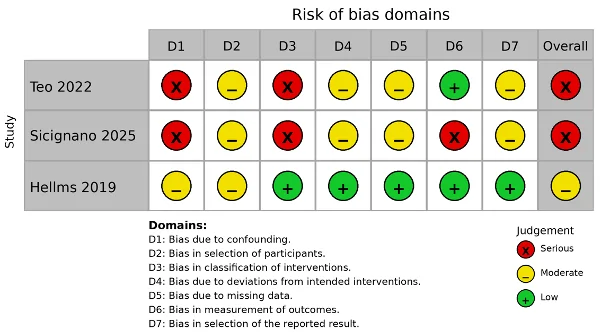
Renal outcome risk of bias for the non-randomised studies (ROBINS-I). Domains: D1, confounding; D2, selection of participants; D3, classification of interventions; D4, deviations from intended interventions; D5, missing data; D6, measurement of the renal outcome; D7, selection of the reported result. The Overall column shows the renal outcome rating. Green, low; yellow, moderate; red, serious [68,72,73].

The domain-level judgements are displayed as traffic-light plots for the randomised (Figure 2) and the non-randomised (Figure 3) studies.

### 3.3. Characteristics of Included Studies

Eighteen studies were included. Five formed the renal-risk stratum: one CKD cohort [72], one real-world observational cohort [73], two pooled analyses reporting renal-risk subgroups [74,76], and one post-hoc subgroup analysis [70]. The remaining 13 formed the comparative stratum, which was predominantly comprised of randomised trials of diclofenac formulations for osteoarthritis [51,52,62,63,64,65,67,71]. Sample sizes ranged from 40 participants to over 500,000. Topical diclofenac was most commonly given as a 1.5% diclofenac–DMSO solution; gels, patches, and epolamine or diethylamine salts were also represented, and one cohort involved mixed topical NSAID exposure. Designs, agents, comparators, and the renal data reported by each study are given in Table 4, with the complete renal dataset provided as Supplementary Material.

The renal-risk stratum comprised five reports. A retrospective cohort of 6298 adults with chronic kidney disease (mean estimated glomerular filtration rate 41.9 mL/min/1.73 m^2^) compared topical NSAIDs, systemic NSAIDs and no NSAID exposure, with incident KDIGO-defined acute kidney injury within 30 days as the primary outcome; topical exposure was predominantly ketoprofen plaster (71%), diclofenac accounting for a minority of treated patients [72]. A real-world analysis of 521,593 incident users of diclofenac 1% gel identified renal-risk, diabetic and older-adult subgroups at baseline, but included no comparator group and ascertained renal outcomes by diagnostic coding rather than laboratory measurement [73]. A pooled safety analysis of seven phase III osteoarthritis trials contributed 280 participants aged ≥75 years, of whom 138 received topical diclofenac solution [74]; a pooled analysis of five vehicle-controlled gel trials (*n* = 2209) reported adverse events categorically across age and comorbidity subgroups [76]; and a post-hoc analysis of an open-label safety programme (*n* = 947) assessed adverse events by age and comorbidity subgroup without reporting renal laboratory parameters [70]. Because diclofenac accounted for a minority of topical exposure in the principal cohort, estimates from that cohort are carried through the synthesis as topical NSAID class effects rather than as diclofenac-specific estimates (Section 3.4, Section 3.9 and Section 4.4).

### 3.4. Renal Outcomes in Populations at Increased Renal Risk (Primary Synthesis)

The clinical relevance of this stratum is substantial. CKD affects an estimated 788 million adults worldwide [77], the majority of whom report regular use of NSAIDs [78], and concomitant exposure to the nephrotoxic “triple-whammy” combination is frequently observed in contemporary cohorts [79]. Topical diclofenac is recommended preferentially over oral formulations in such patients, making the renal safety evidence in this population particularly relevant.

Five of the 18 included studies reported renal safety data in populations defined by or enriched for increased renal risk. One cohort was restricted to CKD [72]. A large claims-based study identified substantial renal-risk subgroups, including 145,311 patients with baseline renal risk factors, over 106,000 with diabetes, and approximately 200,000 aged ≥65 years [73]. Three additional reports evaluated renal-risk subgroups within trial populations, including participants aged ≥75 years [74], combined hypertensive, diabetic, and cardiovascular subgroups across five gel trials [76], and an open-label cohort stratified by age and comorbidity status [70]. Collectively, these studies encompass very large renal-risk populations and constitute the primary evidence base for this stratum.

**Figure 4:**
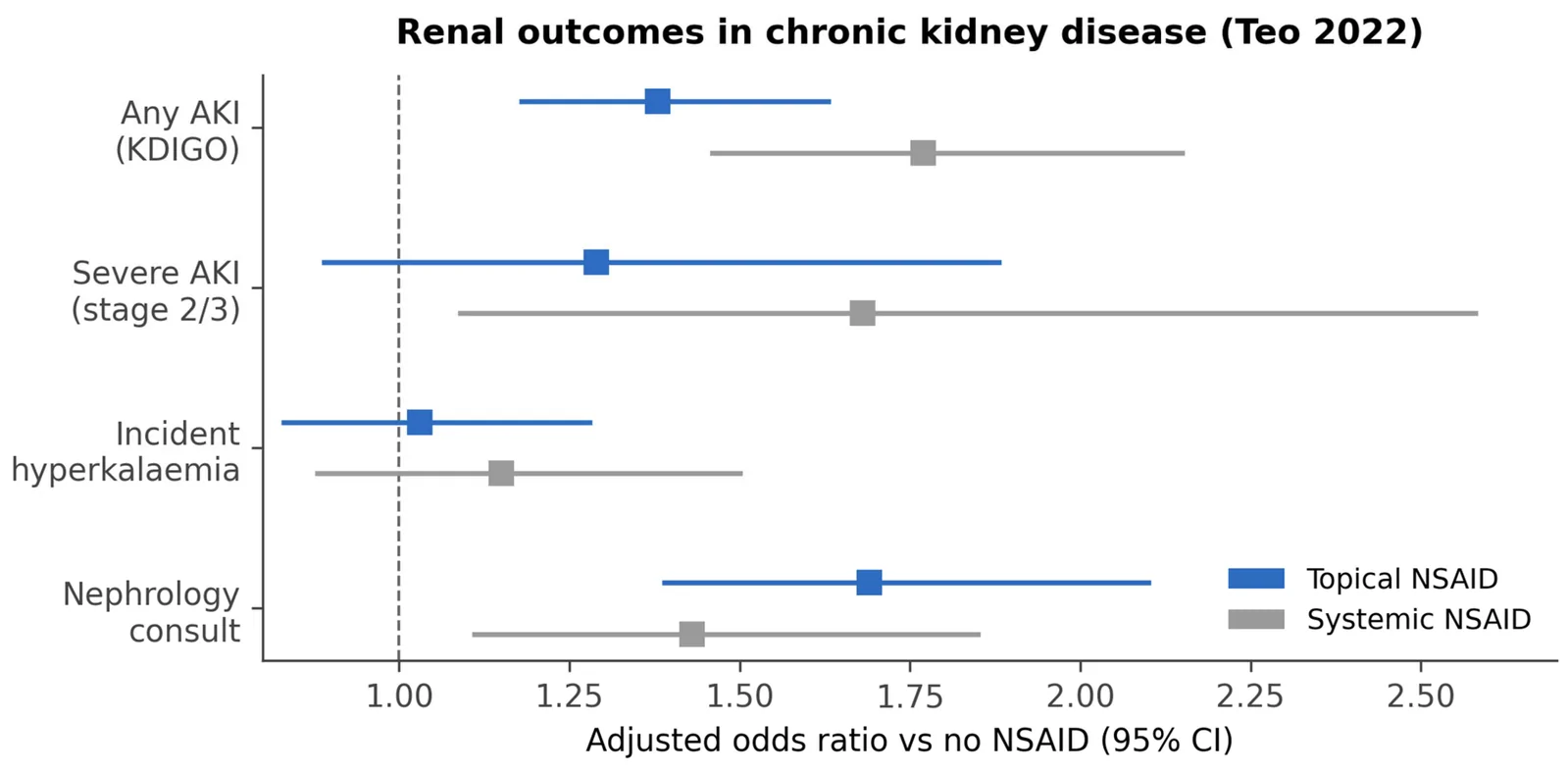
Adjusted odds ratios for renal outcomes in CKD, topical versus systemic NSAIDs, each relative to no NSAID (Teo et al. [72]). Squares are point estimates; horizontal lines are 95% confidence intervals; the dashed line marks an odds ratio of 1.0. Topical NSAIDs were associated with any AKI and with nephrology consultation, but not with severe AKI or hyperkalaemia, for which the confidence interval crossed 1.0. The topical arm was ketoprofen-predominant, so the estimates reflect a topical NSAID class effect and not a diclofenac-specific one.

Within these data, the CKD cohort described in Section 3.3 provided the only adjusted estimates for a clinically defined renal outcome. Topical NSAID exposure was associated with incident AKI within 30 days (adjusted odds ratio 1.38, 95% CI 1.18–1.63) compared with no NSAID exposure. Systemic NSAIDs showed a higher risk (adjusted odds ratio 1.77, 95% CI 1.46–2.15), indicating a consistent route gradient favouring topical administration. For KDIGO stage 2–3 AKI, the association for topical NSAIDs was not statistically significant (adjusted odds ratio 1.29, 95% CI 0.89–1.88), whereas the systemic association persisted. Hyperkalaemia was not increased with either exposure route (topical adjusted odds ratio 1.03, 95% CI 0.83–1.28), and nephrology consultation rates were higher in the topical group (adjusted odds ratio 1.69, 95% CI 1.39–2.10) [72]. In absolute terms, this corresponds to an excess of 4.4% (95% CI 2.3–6.5) in any-stage AKI over 30 days, or one additional event for every 23 patients with CKD exposed (95% CI 15–44) [72] (Figure 4).

**Figure 5:**
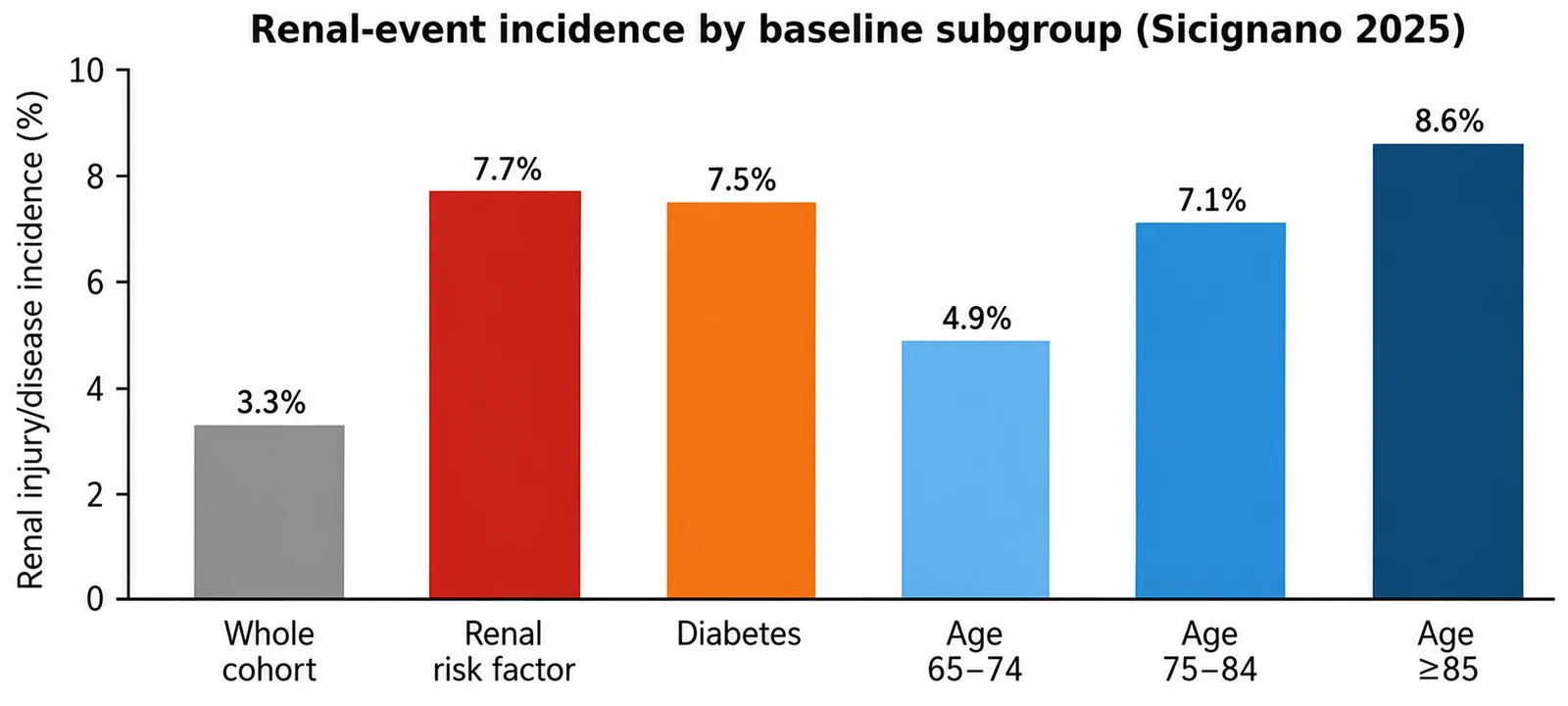
Incidence of the ICD-coded renal composite by baseline subgroup in a single-arm cohort of incident topical diclofenac users (*n* = 521,593) (Sicignano et al. [73]). Incidence was higher in the renal-risk-factor, diabetic, and older subgroups than in the whole cohort and rose with age. The outcome was code-based and not laboratory-confirmed, and 55.4% of the renal-event cohort had concomitant oral NSAID exposure.

The remaining renal-risk studies provided categorical outcomes consistent with these findings. A single-arm claims-based cohort of incident diclofenac gel users reported a renal composite incidence of 3.3% across more than 500,000 records, with higher event rates in renal-risk-factor, diabetic, and older subgroups [73] (Figure 5). A pooled analysis of participants aged ≥ 75 years showed no meaningful differences in serum creatinine changes between topical solution, placebo, and vehicle groups [74]. A pooled analysis of five gel trials reported no excess renal or cardiovascular adverse events across older, hypertensive, diabetic, or cardiovascular subgroups [76]. A post-hoc analysis of an open-label extension reported higher frequencies of peripheral oedema in patients with type 2 diabetes (5.0%) and cardiovascular disease (3.1%), without reporting renal laboratory data for these subgroups [70]. No continuous measure of renal function change was reported in any renal-risk population [70,72,73,74,76]. Evidence was therefore limited to categorical or administrative endpoints rather than laboratory-based renal trajectories.

No study in this stratum was designed to estimate diclofenac-specific effects on renal function in patients at increased renal risk [70,72,73,74,76].

### 3.5. Renal Outcomes in Populations Without Increased Renal Risk (Comparative and Mechanistic Evidence)

The 13 studies in this group did not enrol patients selected for renal risk; most were osteoarthritis trials that excluded clinically active renal disease at baseline, along with two mechanistic studies conducted in healthy volunteers [66,68]. They are presented as comparative and mechanistic evidence rather than as direct estimates in at-risk populations.

**Figure 6:**
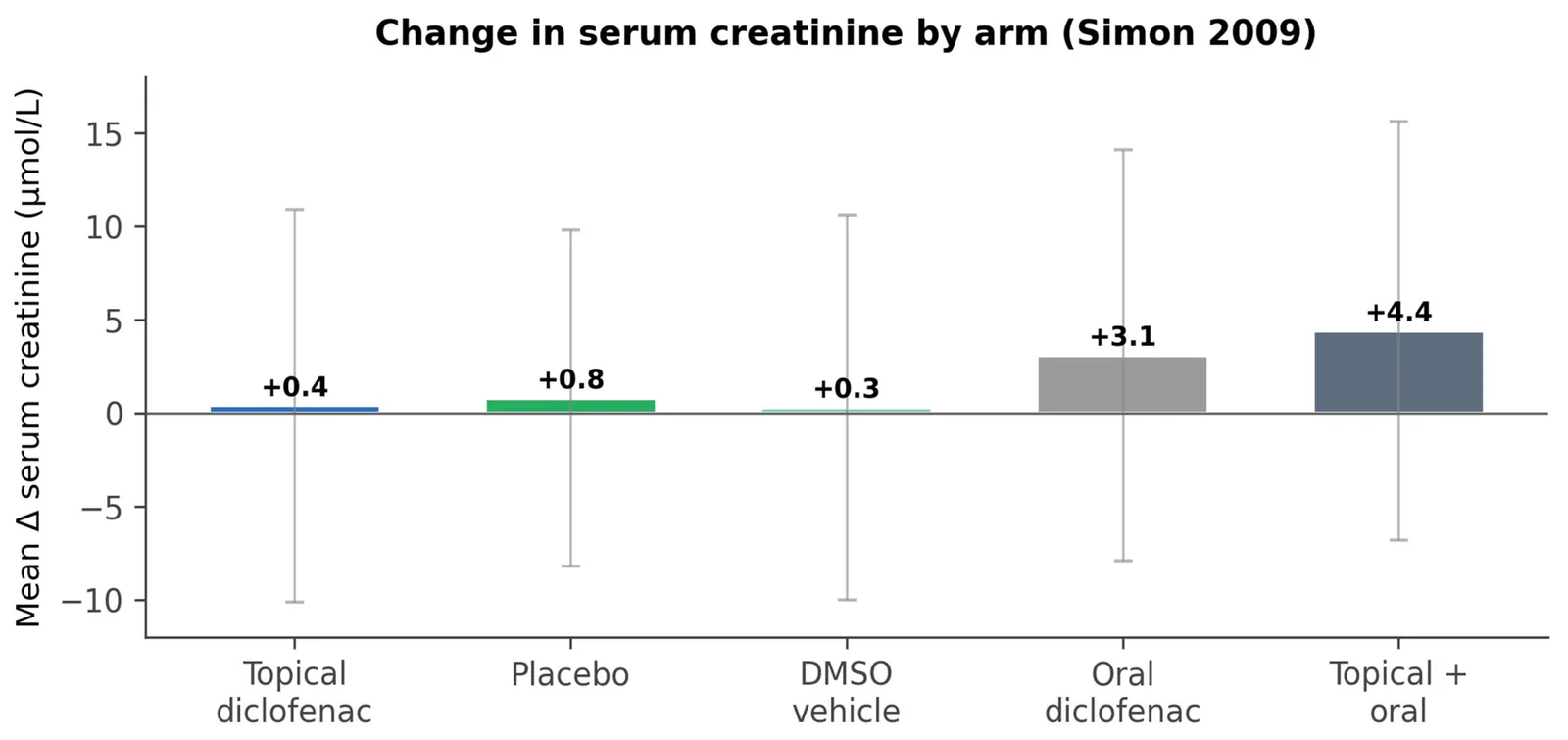
Mean change in serum creatinine by treatment arm in a five-arm trial in knee osteoarthritis (Simon et al. [52]). The topical diclofenac change (+0.4 µmol/L) was comparable to placebo (+0.8) and to the DMSO vehicle (+0.3), whereas the oral (+3.1) and combination (+4.4) arms changed more. Error bars are standard deviations; the population excluded patients at increased renal risk.

**Figure 7:**
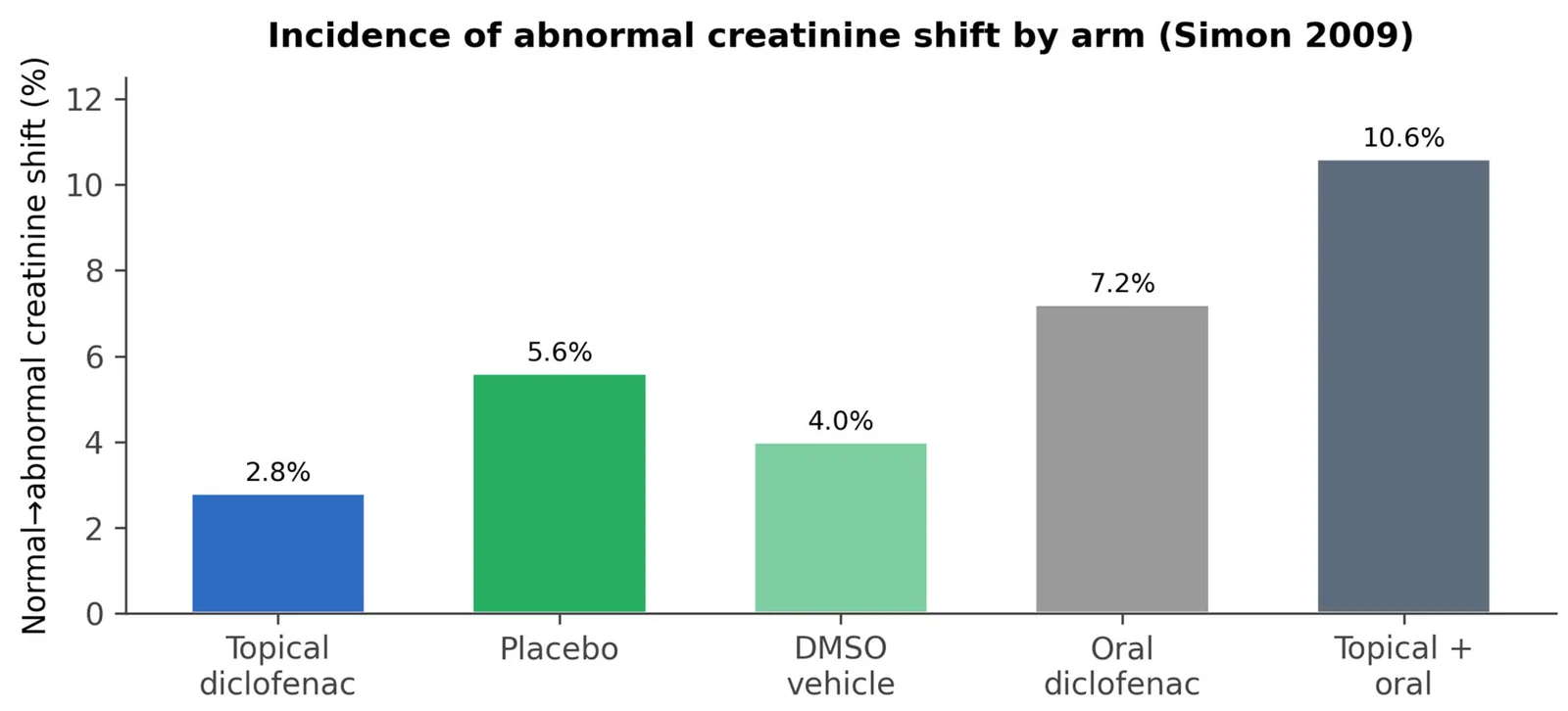
Incidence of a normal-to-abnormal serum creatinine shift by treatment arm (Simon et al. [52]). The overall gradient ran from the topical diclofenac arm (2.8%) to the combination arm (10.6%), consistent with a dose-additive systemic effect; the ordering was not strictly monotonic across the placebo and vehicle arms, as expected for low event counts.

Most quantitative renal data in this group were derived from head-to-head comparisons with oral diclofenac. In an equivalence trial, the topical solution was associated with a minimal mean change in serum creatinine (+0.3 µmol/L) compared with oral diclofenac (+3.3 µmol/L), alongside corresponding differences in creatinine clearance (+0.6 versus −2.7 mL/min) and fewer abnormal laboratory shifts (4% versus 10%) [51]. A five-arm randomised trial reproduced this pattern: changes with topical solution, placebo, and vehicle were comparable, whereas oral and combination therapy produced larger changes and higher proportions of abnormal values (Figure 6 and Figure 7) [52]. Creatinine clearance followed the same ordering across arms. Two pooled analyses of these trials confirmed consistent findings, reporting near-null serum creatinine changes for topical diclofenac compared with both oral diclofenac and placebo [53,75].

The remaining trials assessed renal safety only in qualitative terms. A vehicle-controlled trial reported no renal adverse events in either group, with a small non-significant difference in oedema incidence between arms (2.4% versus 1.2%) [65]. A three-arm trial collected blood and urine samples but reported only the absence of serious renal events without numerical data [63]. Placebo-controlled gel trials reported renal outcomes as unremarkable without presenting laboratory values [62,64]. In perioperative and comparative topical studies, serum creatinine was measured within treatment arms without clinically meaningful between-group differences [67,71]. In one perioperative study, a small within-arm increase in creatinine followed percutaneous nephrolithotomy and was most plausibly attributable to surgical effects rather than treatment exposure [67]. An open-label extension included serial blood sampling over one year but did not report renal laboratory outcomes [69].

Only one mechanistic study directly assessed renal physiology. Using arterial spin-labelling magnetic resonance imaging, renal perfusion decreased after diclofenac exposure, while serum creatinine remained within normal limits during short-term follow-up [68]. This study evaluated a controlled diclofenac challenge rather than sustained topical administration and was the only included investigation to assess a functional renal endpoint [68].

### 3.6. Synthesis by Outcome

Across both study groups, renal outcomes were concentrated in a limited set of endpoints. AKI defined by KDIGO criteria was reported in a CKD cohort, which observed a modest increase associated with topical NSAID exposure that did not extend to severe stages of injury [72]. Continuous serum creatinine change was reported only in general population trials, where the topical solution showed changes comparable to placebo and consistently smaller than those observed with oral diclofenac [51,52]. Creatinine clearance followed the same pattern in the two trials reporting this endpoint [51,52]. Incident hyperkalaemia was assessed only in the CKD cohort and was not increased with topical or systemic exposure [72]. Peripheral oedema was reported intermittently across studies and showed at most small, non-significant differences between groups [65,70]. No study in either population stratum reported proteinuria, albumin-to-creatinine ratio, cystatin C, or tubular injury biomarkers. Renal perfusion was evaluated in a single mechanistic study under short-term experimental conditions [68].

Vote-counting by direction of effect was applicable only to serum creatinine change, the sole outcome reported in at least three studies with comparable data. All available comparisons indicated minimal change with topical diclofenac relative to oral administration [51,52]. Extending the count to categorical creatinine outcomes, five studies showed no worse renal outcome with the topical route than with the comparator [51,52,62,63,64]; under a sign test, this corresponds to a two-sided p-value of 0.06. For all other outcomes, the number of comparable studies was below the prespecified threshold, and findings were reported narratively.

The included evidence encompassed heterogeneous topical formulations, including diclofenac 1.5% dimethyl sulfoxide solution, diclofenac 1% gel, and diclofenac epolamine or diethylamine preparations, which differ in percutaneous absorption characteristics [51,52,65]. The observed consistency in direction of effect should therefore be interpreted across formulations rather than as a product-specific quantitative estimate. The results are therefore presented as study-level descriptive summaries (Section 2.5). Where only relative measures were reported, absolute values were derived from published event counts to support interpretation.

In the single adjusted cohort within the renal-risk stratum, any-stage AKI occurred in 380 of 2014 patients exposed to topical NSAIDs (18.9%) and in 468 of 3230 unexposed patients (14.5%); the corresponding absolute risk difference and number needed to harm are reported in Section 3.4 [72]. Topical and systemic NSAIDs were each compared against no NSAID exposure within the same cohort, allowing an indirect within-study comparison: the ratio of adjusted odds ratios for topical versus systemic exposure was 0.78 (95% CI 0.61–1.00) [72].

Low- and moderate-risk studies were predominantly in general population settings, whereas renal-risk cohorts were more frequently rated at higher risk of bias (Section 3.2, Table 1).

### 3.7. Overlapping Populations

Shared participants were identified across several reports and accounted for before synthesis. Table 3 pairs each pooled analysis with its source trials. Further overlap existed between the seven-trial pooled analyses [74,75] and the vehicle-controlled solution trials, including Roth et al. 2004 [65], and between the post-hoc subgroup analysis and the open-label extension it re-analysed [69,70]. The CKD cohort was derived from a broader inappropriate-prescribing dataset [72]. For each overlap cluster, a single dataset representation was retained, as specified in Section 3.2.

### 3.8. Comparison Across Populations

**Figure 8:**
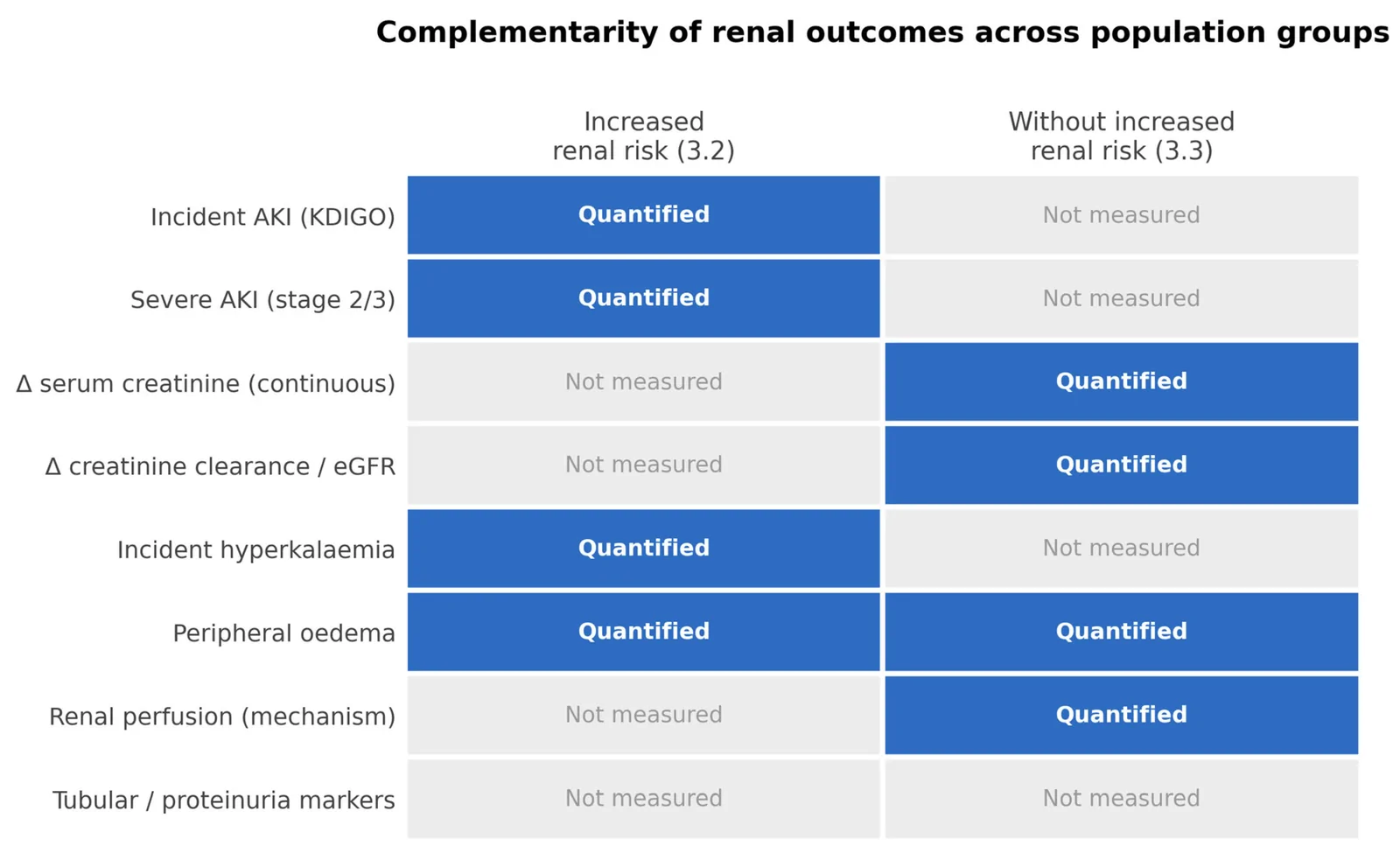
Complementarity of renal outcomes across the two population groups. Cells are shaded where at least one study in the group reported the outcome as a numeric value or estimate (quantified) and left pale where no study in the group measured it. The pattern is close to a mirror image: AKI and hyperkalaemia were quantified only in the renal-risk group, whereas continuous renal function measures and renal perfusion were quantified only in the group without increased renal risk. Peripheral oedema was quantified in both, and tubular or proteinuria markers in neither.

The two population groups assessed different outcomes using different methodologies and did not share participants; accordingly, the results were compared qualitatively rather than pooled. The renal outcomes reported in each group are listed in Table 5 and displayed in Figure 8.

#### 3.8.1. Convergence

**Figure 9:**
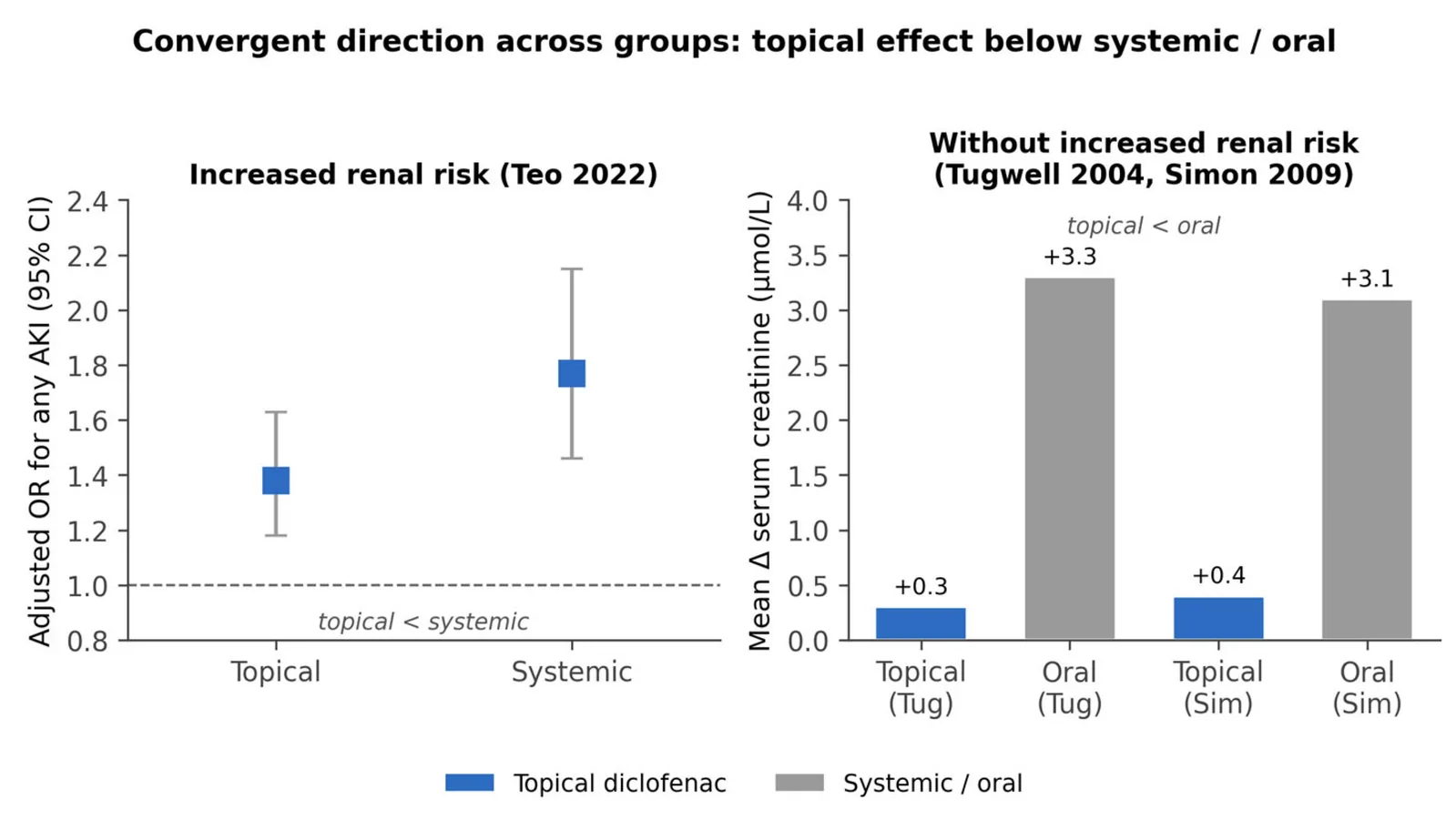
Convergent direction of effect across the two groups, shown on separate axes because the measures are not directly comparable. In the renal-risk cohort (**left**), the adjusted odds of any AKI were lower for the topical than for the systemic route (Teo et al. [72]). In the general population trials (**right**), the mean rise in serum creatinine was smaller with the topical route than with oral diclofenac (Tugwell et al. [51] and Simon et al. [52]). Both groups place the topical effect below systemic or oral exposure.

Across comparable endpoints, both groups showed consistent directionality (Figure 9). Head-to-head trials in general populations reported smaller changes in serum creatinine with the topical route compared with oral diclofenac [51,52], while the renal-risk cohort showed a lower adjusted odds of AKI for topical versus systemic NSAIDs (1.38 vs. 1.77) [72]. Neither group demonstrated a statistically significant increase in severe AKI or hyperkalaemia [72], and controlled trials reported minimal or no renal adverse events [65,74]. Peripheral oedema was infrequent and of low magnitude where reported in both populations [65,70].

#### 3.8.2. Divergence and Complementarity

Outcome coverage differed between the strata, as set out in Table 5: clinical events (KDIGO-defined AKI and hyperkalaemia) were quantified only in the renal-risk cohort [72], continuous renal function measures only in general population trials [51,52], and renal perfusion in a single mechanistic study [68]. The two strata are therefore complementary rather than comparable, covering different points on the severity spectrum: trials describe renal biomarkers under preserved renal function, and the cohort describes clinical renal events under increased baseline vulnerability.

### 3.9. Certainty of Evidence

Certainty was assessed with the GRADE approach for the main renal outcomes, separately within each population stratum, and is presented in Table 6. Ratings ranged from high to moderate. In the general population stratum, renal outcomes rested on objective laboratory and imaging measurements, derived primarily from randomised trials, several of which were at low risk of bias for the renal endpoint. Change in serum creatinine therefore reached high certainty, with no downgrade for risk of bias, inconsistency, indirectness, or imprecision. The mechanistic study of renal perfusion by arterial spin-labelling MRI provided moderate-certainty evidence for that endpoint, downgraded once for imprecision because of its small sample size. In the renal-risk stratum, AKI and hyperkalaemia were ascertained objectively by KDIGO criteria or coded diagnoses in very large cohorts. Both were rated moderate, with a single downgrade for indirectness of exposure classification, since topical exposure in the principal cohort was predominantly non-diclofenac.

Two methodological considerations informed this assessment. First, GRADE reflects certainty for each outcome rather than overall study quality; while several studies showed limitations in reporting renal outcomes, the key contributing cohorts used objective outcome ascertainment, which is the most relevant domain for certainty in this review. Second, imprecision was minimal in cohort-based outcomes due to large sample sizes, and the only consistent limitation was indirectness of exposure classification rather than random error.

## 4. Discussion

### 4.1. Principal Findings

This systematic review aimed to evaluate whether the renal safety profile commonly attributed to topical diclofenac extends to adults at increased renal risk, in whom prostaglandin-dependent renal autoregulation is most clinically relevant. The findings support a stratified but internally consistent pattern across populations.

In adults without increased renal risk, topical diclofenac demonstrated a favourable renal profile. Changes in serum creatinine and creatinine clearance were consistently comparable to placebo and substantially smaller than those observed with oral diclofenac, based on objective laboratory measurements. Given the inclusion of several trials at low risk of bias for renal outcomes, the certainty of this evidence was rated as high.

In populations at increased renal risk, the available evidence showed a concordant direction of effect, although with greater limitations in data structure and exposure classification. The principal adjusted cohort indicated a modest increase in AKI, predominantly driven by milder events, with consistently lower risk estimates for topical compared with systemic NSAID exposure. This evidence was rated as moderate certainty, with indirectness of topical diclofenac exposure representing the primary limitation.

Topical diclofenac therefore appears more favourable than oral diclofenac in both strata, the difference being constrained by indirect exposure data rather than by inconsistency of effect.

### 4.2. Mechanistic Basis for the Favourable Profile

The favourable renal profile observed in general populations is mechanistically coherent with the known pharmacology of NSAIDs. Renal adverse effects are primarily mediated through inhibition of cyclo-oxygenase–derived prostaglandins, which are essential for maintaining afferent arteriolar vasodilatation under conditions of reduced renal perfusion. The extent of this effect is dependent on systemic drug exposure at the level of the kidney.

Topical administration results in substantially lower systemic exposure compared with equivalent oral dosing, while maintaining higher local tissue concentrations at the site of application. This pharmacokinetic profile reduces systemic cyclo-oxygenase inhibition and, consequently, renal prostaglandin suppression. Consistent with this mechanism, the within-cohort comparison demonstrated a lower relative odds of AKI for topical versus systemic exposure (approximately 0.78 relative to oral exposure) [72]. These data support a pharmacologically plausible relative renal advantage of topical administration driven by reduced systemic exposure, rather than an absence of renal effect.

### 4.3. Insensitivity of the Reported Endpoints

This distinction between relative renal advantage and absolute safety is further informed by the endpoints used across the evidence base. Renal function was assessed predominantly using serum creatinine, estimated glomerular filtration rate, and, in some studies, creatinine clearance. These markers have limited sensitivity for detecting early or predominantly haemodynamically mediated renal injury, as compensatory renal autoregulatory mechanisms may maintain glomerular filtration until substantial functional loss has occurred, and rises in serum creatinine typically lag behind the onset of injury. Accordingly, the absence of detectable changes in serum creatinine should not be interpreted as evidence of no renal effect, but rather as an absence of detectable change using relatively insensitive biomarkers. No included study assessed more sensitive markers such as cystatin C, urinary albumin-to-creatinine ratio, or tubular injury biomarkers such as neutrophil gelatinase-associated lipocalin (NGAL) and kidney injury molecule-1 (KIM-1). Direct assessment of renal perfusion was available in only one mechanistic study, which reported a reduction following diclofenac exposure [68].

The consequences of this measurement gap are greatest in the population this review set out to characterise. Where nephron reserve is already reduced, prostaglandin-dependent autoregulation carries a larger share of glomerular perfusion, so a haemodynamically mediated effect of a given magnitude produces a proportionally greater functional loss [4]. Serum creatinine responds late and only once that loss is substantial, and it does not report tubular injury at all. Evidence assembled from creatinine alone can therefore demonstrate the absence of a detectable functional change, but not the absence of injury.

Closing this gap requires studies in which renal outcomes are the design objective rather than an incidental safety observation. The relevant design is a prospective cohort or randomised trial recruiting patients with CKD G3–G4 and other renal-risk phenotypes, with exposure ascertained at the level of the diclofenac product—formulation, applied dose, treated surface area, and duration—rather than as topical NSAID use; protocolised sampling at baseline and at fixed intervals during and after exposure; and an outcome panel extending beyond serum creatinine to cystatin C, albumin-to-creatinine ratio, and markers of tubular injury. Follow-up would need to extend beyond the treatment period to establish whether any acute change resolves or contributes to a longer-term decline in renal function.

Whether this measurement gap undermines the certainty ratings requires the distinction that GRADE itself draws. Certainty is assigned to an outcome rather than to a construct: the high rating attaches to the finding that topical diclofenac produces little to no change in serum creatinine, an endpoint measured directly, consistently and by objective methods. It does not extend to subclinical tubular or early glomerular injury, which no included study measured and for which no certainty rating can be assigned. The biomarker gap therefore does not lower confidence in the conclusions drawn about the measured endpoints; it delimits their scope. The absence of sensitive biomarkers does, however, limit confidence in concluding renal safety with respect to subclinical kidney injury, which the available evidence can neither demonstrate nor exclude. Read strictly, the evidence supports the statement that topical diclofenac does not detectably alter conventional measures of glomerular filtration, and it does not support the broader statement that topical diclofenac is free of renal injury.

### 4.4. The Signal in Patients at Risk and the Undocumented High-Risk Scenario

Findings in the renal-risk stratum warrant explicit reporting rather than attenuation. In the only adjusted cohort, exposure to topical NSAIDs was associated with an increased risk of any-stage AKI (adjusted odds ratio 1.38) [72].

Two factors should be considered when interpreting this signal. First, the association was not observed for more severe KDIGO stage 2–3 AKI, which was not statistically significant. Second, exposure classification was heterogeneous: 71% of topical exposure in that cohort was ketoprofen plaster, with topical diclofenac representing a minority of treated patients, so the estimate is properly read as a topical NSAID class effect rather than a diclofenac-specific one [72]. The estimate is therefore indirect for topical diclofenac, although the direction of effect remains consistent with increased risk in this population.

Beyond exposure classification, the observational architecture of this evidence imposes further interpretive limits. Both contributing cohorts were rated at serious risk of bias, with confounding the dominant domain. In patients with CKD, the decision to prescribe a topical rather than a systemic NSAID is itself informed by the prescriber’s assessment of renal vulnerability, so confounding by indication, also termed indication bias, would be expected to operate in the direction of harm and is not removed by adjustment for measured covariates. Differential ascertainment may act in the same direction, in that the topical group had higher rates of nephrology consultation [72] and closer renal surveillance would increase the detection of mild, laboratory-defined events. The observed pattern, an association with any-stage AKI but not with KDIGO stage 2–3 AKI, is compatible with such an effect, though equally compatible with a genuine but small haemodynamic signal that does not progress. The within-cohort comparison of topical against systemic exposure is not exempt, since the two routes are channelled to different patients. The second cohort was single-arm and permitted no control of confounding at all, and concomitant oral NSAID exposure was recorded in 55.4% of its renal-event cohort [73], so its incidence estimates cannot be attributed to topical diclofenac alone.

An additional limitation relates to clinical context. Concomitant use of the “triple-whammy” combination is frequent in older patients with CKD, in whom prostaglandin-dependent renal autoregulation is particularly vulnerable [79]. Despite this clinical relevance, no included study specifically evaluated topical diclofenac in patients receiving this combination. As a result, the highest-risk prescribing scenario remains largely unaddressed by the current evidence base.

### 4.5. Implications for Clinical Practice and Guidelines

These findings support rather than challenge the current preference for topical over oral NSAIDs in patients at increased renal risk. OARSI and American College of Rheumatology recommendations position topical NSAIDs as first-line therapy for knee osteoarthritis and favour them where systemic exposure should be minimised [20,21], with concordant national and population-specific guidance for older adults [22,23]. The route gradient observed here is consistent with that positioning: within a single CKD cohort, adjusted odds of AKI were lower for topical than for systemic exposure [72], and in general populations serum creatinine changes with topical diclofenac were comparable to placebo and smaller than with oral diclofenac [51,52].

What the evidence does not support is the stronger inference that topical application removes renal risk where renal reserve is reduced. For patients with an eGFR of 30–60 mL/min/1.73 m^2^, the only adjusted estimate available indicates one additional any-stage AKI event for every 23 patients exposed over 30 days—an increment that is modest but not negligible, and one that should be weighed against the analgesic benefit obtained and against the renal risk of the alternatives, whether oral NSAIDs, for which the same cohort recorded a higher estimate [72], or the analgesic options that remain available in CKD [45]. For short-term treatment of a localised musculoskeletal complaint, that balance is consistent with the guideline preference for the topical route. It is less clearly established where treatment is prolonged, where large surface areas are treated, or where concurrent prescription of a renin–angiotensin system inhibitor and a diuretic reconstitute the “triple-whammy” configuration [43,44,79], a scenario for which no included study provides direct evidence.

The two-stratum structure also determines how the reassuring general population findings should be read at the bedside. Those findings establish a property of the drug rather than of the patient: for the same indication, the topical route delivers substantially less systemic exposure than the oral route and produces a correspondingly smaller change in measured renal function [51,52]. That premise is transportable to patients at increased renal risk, and it is what justifies preferring the topical route in them. What is not transportable is the absolute reassurance, because the magnitude of a prostaglandin-dependent haemodynamic effect depends on the renal reserve of the individual receiving it, and the trials that generated the reassuring estimates excluded the patients in whom that reserve is reduced. The comparative reference stratum should therefore inform the choice between routes, whereas the absolute renal risk for an individual patient with CKD rests on the renal-risk stratum, where the evidence is indirect and the only adjusted estimate remains compatible with a modest excess of any-stage AKI [72].

One qualification should accompany any translation of these findings into practice: guideline recommendations address the sustained use that osteoarthritis frequently entails, whereas the renal outcome data available to support them are confined to short observation windows.

### 4.6. Limitations

Several limitations should be considered when interpreting these findings. First, the synthesis included heterogeneous study designs, topical formulations, comparators, and patient populations. This heterogeneity precluded meta-analysis and necessitated a structured narrative synthesis without statistical pooling, but it nevertheless limits the strength of cross-study inference. Second, the primary renal-risk stratum was based largely on observational cohort data, which were subject to inherent limitations, including indirect exposure classification and variability in outcome ascertainment. As a result, estimates for the population of greatest clinical relevance should be interpreted with appropriate caution. Third, the absolute and indirect effect estimates presented in this review were derived as descriptive recalculations from individual studies rather than pooled analyses. Consequently, they reflect the limitations of the source datasets and should not be interpreted as independent summary effect estimates. Fourth, renal outcomes were ascertained over short observation windows. In the only adjusted cohort, incident AKI was defined within 30 days of exposure [72]; the single mechanistic study assessed renal perfusion under short-term conditions [68]; and the one included study with a one-year observation period collected renal laboratory data but did not report them [69]. No included study described the trajectory of renal function beyond the treatment period. Whether repeated or prolonged topical diclofenac use influences longer-term renal outcomes, including progression of CKD, therefore remains unestablished. Fifth, two constraints applied to the literature search itself. CENTRAL was not searched directly because of institutional access limitations, and this was only partially mitigated by the overlap between the four databases searched and by consultation of ClinicalTrials.gov and the WHO ICTRP; incomplete retrieval of trial records can therefore not be excluded. In addition, the search strategies were checked internally against the PRESS 2015 guideline but did not undergo formal external peer review by an information specialist, which was not feasible, so residual errors in translation across databases cannot be excluded.

### 4.7. Strengths and Limitations of the Evidence Base

The overall certainty of the synthesised evidence was judged to be moderate to high; the distribution of renal outcome risk of bias is given in Section 3.2 and Table 1. The main limitations across the evidence base related less to the conduct of the studies than to the measurement, ascertainment, and reporting of renal outcomes.

A recurring methodological issue was the collection of renal laboratory data that were subsequently described only qualitatively as showing no clinically meaningful changes, without publication of the underlying numerical results. This reporting pattern was considered suggestive of potential selective non-reporting and was therefore incorporated into the risk-of-bias assessment as a concern related to missing outcome data. Its direction cannot be resolved from the published record: omission of non-significant findings inflates apparent uncertainty, while small but measurable renal changes may equally have gone unreported.

Interpretation is further constrained by the structure of the evidence: renal risk is estimated from observational cohorts with indirect exposure classification, mixed topical NSAID formulations, and coded rather than laboratory-based outcomes, whereas the trials providing continuous renal function data excluded patients with clinically significant renal impairment.

The applicability of this evidence to nephrology practice therefore requires explicit statement. The randomised trials contributing continuous renal function data enrolled ambulatory patients with osteoarthritis and excluded clinically active or clinically significant renal disease at baseline; none reported enrolment of patients with heart failure or acute intercurrent illness, so these trials characterise renal behaviour under preserved reserve rather than renal safety in nephrology populations. The review protocol itself excluded CKD G5, dialysis-dependent disease and active AKI, while decompensated cirrhosis and nephrotic syndrome were specified only as sensitivity analysis conditions rather than primary eligibility criteria (Section 2.2). The population for which any adjusted estimate exists is therefore ambulatory CKD G3–G4, represented by a single cohort in which 79% of participants were stage G3 and 21% stage G4 [72]. For advanced CKD, dialysis-dependent disease, acute illness and the concurrent prescribing configurations that dominate inpatient nephrology, the evidence reviewed here is silent, and practice in those settings continues to rest on pharmacological reasoning and precautionary guidance [45] rather than on outcome data.

The scope of renal assessment was also limited, as set out in Section 4.3.

The evidence base also varies with respect to study provenance. A substantial proportion of the available safety data originates from manufacturer-sponsored clinical development programmes, whereas evidence concerning patients at elevated renal risk is derived largely from observational research. Although these differences are transparently reported in the conflict-of-interest disclosures of the included studies, they remain relevant when interpreting the consistency and generalisability of the findings.

These limitations do not invalidate the consistency observed across study designs, but they do warrant cautious interpretation; the study design required to resolve them is specified in Section 4.3.

## 5. Conclusions

Topical diclofenac demonstrates a consistent pattern of renal safety across available evidence, with interpretation stratified by population risk. In adults without increased renal risk, the evidence is robust and of high certainty, showing little to no change in serum creatinine and consistently smaller renal effects compared with oral diclofenac under objective laboratory assessment.

In adults with increased renal risk, the evidence is of moderate certainty and is based primarily on observational data and subgroup analyses. These suggest a modest increase in AKI with topical NSAID exposure overall, while also indicating a consistently lower relative risk compared with systemic NSAID use. However, the available data are indirect for diclofenac specifically and do not include systematic measurement of sensitive renal biomarkers.

Across both populations, the direction of effect is broadly consistent and favours topical over systemic administration with respect to renal outcomes, but the magnitude of benefit in high-risk patients remains uncertain due to exposure heterogeneity and limited granularity of renal measurements. Further prospective studies focusing specifically on topical DCF in patients with CKD, incorporating standardised laboratory endpoints and contemporary nephrotoxicity biomarkers, are required to refine these estimates and strengthen certainty in the high-risk population.

## Figures and Tables

**Table 1:** Distribution of renal outcome risk of bias across the 14 studies given a primary appraisal. The four pooled analyses are derived and excluded from this count.

Renal Outcome Risk of Bias	*n*	Studies
Low	3	Altman 2009 [62], Bookman 2004 [63], Niethard 2005 [64]
Some concerns/Moderate	8	Tugwell 2004 [51], Simon 2009 [52], Roth 2004 [65], Kienzler 2010 [66], Baheti 2025 [67], Hellms 2019 [68], Peniston 2011 [69], Peniston 2012 [70]
Serious/High	3	Ahmadabadi 2020 [71], Teo 2023 [72], Sicignano 2025 [73]

**Table 2:** Renal outcome appraisal of the single-arm studies (JBI case series checklist).

Study	Inclusion Clear	Complete Inclusion	Valid Renal Measurement	Complete Follow-Up	Analysis Appropriate	Overall (Renal)
Peniston et al. 2011 [69]	Yes	Partial	Partial	No	Partial	Partial
Peniston et al. 2012 [70]	Yes	Partial	Partial	No	No	Partial

**Table 3:** Pooled analyses with derived renal risk of bias and the de-duplication rule applied at synthesis.

Analysis	Source Trials	Renal RoB (Derived)	De-Duplication
Roth et al. 2011 (*J. Pain Res.*) [53]	Tugwell 2004 [51] + Simon 2009 [52]	Moderate	Use the two source trials or this pool, not both.
Roth et al. 2012 (*Clin. Interv. Aging*) [74]	Seven solution-vs-vehicle trials	Serious	Elderly subset; overlaps the source trials.
Roth et al. 2011 (*Postgrad. Med.*) [75]	Seven solution-vs-vehicle trials	Moderate	Placebo-controlled pool; overlaps the source trials.
Baraf et al. 2012 [76]	Five gel-vs-vehicle trials	Serious	Overlaps Altman 2009 [62] and the gel pool.

**Table 4:** Characteristics of the 18 included studies. AE, adverse event; AKI, acute kidney injury; CKD, chronic kidney disease; CrCl, creatinine clearance; CV, cardiovascular; fMRI-ASL, arterial spin-labelling magnetic resonance imaging; KDIGO, Kidney Disease: Improving Global Outcomes; RCT, randomised controlled trial; RWE, real-world evidence; sCr, serum creatinine. The two pooled analyses by Roth et al. (2011), Roth et al. (2012), and Baraf et al. (2012) overlap their source trials and were not double-counted (Section 3.7) [53,74,76].

Study	Design	Group	Topical Agent	Comparator	Renal Data Reported
Teo et al.2023 [72]	Retrospective CKD cohort	At-risk (L1)	Mixed topical NSAID (ketoprofen-predominant)	No-NSAID; systemic NSAID	Incident AKI (KDIGO), hyperkalaemia, nephrology consult
Sicignano et al. 2025 [73]	Retrospective RWE cohort	At-risk (L2)	Diclofenac 1% gel	None (single-arm)	ICD-coded renal composite (incidence)
Roth et al. 2012 [74]	Pooled 7 RCTs (≥75 y subset)	At-risk (L2)	Diclofenac–DMSO solution	Placebo; vehicle	Δ serum creatinine (subgroup)
Baraf et al. 2012 [76]	Pooled 5 RCTs (subgroups)	At-risk (L2)	Diclofenac 1% gel	Vehicle gel	Renal/CV AE incidence (subgroups)
Peniston et al. 2012 [70]	Post-hoc single-arm subgroups	At-risk (L2)	Diclofenac–DMSO solution	None	Categorical AE (oedema by subgroup)
Tugwell et al. 2004 [51]	RCT, double-dummy equivalence	General (L4)	Diclofenac–DMSO solution	Oral diclofenac	Δ sCr, Δ CrCl (head-to-head)
Simon et al. 2009 [52]	RCT, 5-arm	General (L4)	Diclofenac–DMSO solution	Oral; placebo; vehicle	Δ sCr, Δ CrCl (by arm)
Roth et al. 2004 [65]	RCT, vehicle-controlled	General (L4)	Diclofenac–DMSO solution	Vehicle	Renal AE composite (qualitative)
Bookman et al. 2004 [63]	RCT, 3-arm	General (L4)	Diclofenac–DMSO solution	Vehicle; placebo	Renal AE (qualitative)
Altman et al. 2009 [62]	RCT, placebo-controlled	General (L4)	Diclofenac 1% gel	Vehicle gel	Laboratory narrative only
Niethard et al. 2005 [64]	RCT, placebo-controlled	General (L4)	Diclofenac diethylamine gel	Placebo gel	Laboratory narrative only
Baheti et al. 2025 [67]	RCT, two-arm (perioperative)	General (L4)	Diclofenac patch	Ketoprofen patch	Δ sCr (within-arm)
Ahmadabadi et al. 2020 [71]	RCT, two-arm	General (L4)	Diclofenac gel	Zingiber zerumbet ointment	Δ sCr, BUN (within-arm)
Peniston et al. 2011 [69]	Open-label extension	General (L4)	Diclofenac–DMSO solution	None	Renal labs collected, not reported
Hellms et al. 2019 [68]	Interventional imaging	General (L4)	Topical vs. oral diclofenac	Oral diclofenac	Renal perfusion (fMRI-ASL); qualitative creatinine
Kienzler et al. 2010 [66]	RCT, 3-way crossover (pharmacokinetic)	General (L4)	Diclofenac 1% gel	Oral diclofenac	Renal AE composite (qualitative)
Roth et al. 2011 (JPR) [53]	Pooled 2 RCTs	General (L4)	Diclofenac–DMSO solution	Oral diclofenac	Δ sCr (vs oral)
Roth et al. 2011 (PGM) [75]	Pooled 7 RCTs	General (L4)	Diclofenac–DMSO solution	Placebo	Δ sCr (vs placebo)

**Table 5:** Complementarity of renal outcomes across the two population groups. AKI, acute kidney injury; eGFR, estimated glomerular filtration rate; fMRI-ASL, arterial spin-labelling magnetic resonance imaging; NS, not significant.

Renal Outcome	Increased Renal Risk (Section 3.4)	Without Increased Renal Risk (Section 3.5)
Incident AKI (KDIGO)	Reported—topical adj OR 1.38 (Teo et al. [72])	Not measured in any trial
Severe AKI (stage 2/3)	Reported—1.29, NS (Teo et al. [72])	Not measured
Continuous Δ serum creatinine	Not reported (negligible in ≥75 y subset only)	Reported—topical ≈ placebo « oral (Tugwell et al. [51], Simon et al. [52], Roth et al. 2011 [53,75])
Δ creatinine clearance/eGFR	Not reported	Reported—topical ≈ placebo (Tugwell et al. [51], Simon et al. [52]); eGFR unchanged (Hellms et al. [68])
Incident hyperkalaemia	Reported—1.03, NS (Teo et al. [72])	Not reported
Peripheral oedema	Reported—subgroup 3–5% (Peniston et al. 2012 [70])	Reported—2.4%, NS (Roth et al. 2004 [65])
Renal perfusion (mechanism)	Not assessed	Measured once (Hellms et al. [68], fMRI-ASL)
Tubular/proteinuria biomarkers	Not assessed	

**Table 6:** GRADE Summary of Findings for the main renal outcomes, by population stratum. GRADE certainty: high, moderate, low, or very low. Strata A (increased renal risk) and B (general/not at renal risk) are reported separately and were not pooled. Wording follows GRADE informative-statement conventions and is interpreted cautiously: the ratings reflect indirect evidence rather than a demonstration of safety. ASL, arterial spin-labelling; ICD, International Classification of Diseases; KDIGO, Kidney Disease: Improving Global Outcomes; NSAID, non-steroidal anti-inflammatory drug; RCT, randomised controlled trial.

Outcome	Population (Stratum)	Studies (Participants)	What the Evidence Suggests	Certainty (GRADE)	Reasons for the Rating
AKI (KDIGO/ICD-coded)	At increased renal risk (A)	2 non-randomised (≈528,000)	Topical NSAID exposure is probably associated with a small increase in acute kidney injury risk; the outcome was measured objectively in very large cohorts, but the exposure was predominantly a non-diclofenac NSAID.	Moderate	Downgraded once for indirectness (topical exposure in the principal cohort was predominantly a non-diclofenac NSAID). The outcome was ascertained objectively by KDIGO or coded criteria in very large cohorts, which kept imprecision minimal and supported a moderate rather than a lower rating.
Incident hyperkalaemia	At increased renal risk (A)	1 non-randomised (≈6300)	Topical NSAID exposure probably results in little to no difference in incident hyperkalaemia.	Moderate	Downgraded once for indirectness. The large cohort and objective ascertainment limited imprecision and supported the rating.
Change in serum creatinine (continuous)	General/not at increased renal risk (B)	2 RCTs (≈1000) + 1 mechanistic	Topical diclofenac results in little to no change in serum creatinine on objective laboratory testing.	High	Not downgraded. Renal function was measured objectively and reported consistently across trials, several of which were at low risk of bias for the renal outcome; the direction and magnitude were concordant.
Renal perfusion by functional MRI (ASL)	General/healthy volunteers (B)	1 mechanistic (24)	Topical diclofenac probably results in little to no change in renal perfusion on objective imaging.	Moderate	Downgraded once for imprecision (single small mechanistic study). Direct, objective physiological measurement supported the rating.
Renal adverse events (qualitative/composite)	Both strata (descriptive)	≈10 studies	Reported renal adverse events were infrequent and broadly consistent with little to no excess, although often described qualitatively.	Moderate	Downgraded once for the descriptive nature of much of the reporting. Most contributing studies were at low or moderate risk of bias for the renal outcome.

No study assessed early or subclinical renal injury markers, which remains an evidence gap.

## Data Availability

All data analysed in this review are derived from the included published studies and are available within the article and its Supplementary Materials.

## Supplementary Materials

The following supporting information can be downloaded at: https://www.mdpi.com/article/10.3390/medicina62081606/s1, Supplementary Material S1: Search strategy and search results; Supplementary Material S2: Complete renal dataset; Supplementary Material S3: PRISMA 2020 checklist.
