# Supplementary material for: Renal Safety of Topical Diclofenac in Adults with Increased Renal Risk: A Systematic Review
Source: Medicina (Kaunas). 2026 Aug 21;62(8):1606. doi: 10.3390/medicina62081606 (PMC13515200; doi:10.3390/medicina62081606)
Supplement: Supplementary file 1 [file medicina-62-01606-s001.zip › Supplementary Material S1.pdf]

Supplementary Material S1

Search strategy and search results
This file supports the systematic review "Renal Safety of Topical Diclofenac in Adults with Increased Renal Risk: A Systematic Review." It reports the complete, database-specific search strategies and the number of records retrieved at each stage, corresponding to the PRISMA 2020 flow diagram presented as Figure 1 in the main manuscript. The strategies are reported in accordance with the PRISMA-S extension for reporting literature searches (Rethlefsen et al., Syst Rev 2021). The database searches were executed on 18 May 2026 using a predefined search cut-off date of 19 April 2026; therefore, 19 April 2026 represents the definitive search date used for study eligibility.

S1.1. Databases, platforms, and search dates
Four bibliographic databases were searched from inception to the definitive search date: MEDLINE via PubMed, Embase via Embase.com, Web of Science Core Collection, and Scopus. Within each database the three concept blocks (Block 1, diclofenac; Block 2, topical administration; Block 3, renal outcome) were entered as separate query sets and combined with the Boolean operator AND. In Embase, a final step excluded non-human records. The Cochrane Central Register of Controlled Trials (CENTRAL) was not searched directly because of institutional access limitations.

S1.2. Full database-specific search strategies
The complete strategies are reproduced verbatim below. Each block is shown as entered; the final search combined Block 1 AND Block 2 AND Block 3 (with the non-human exclusion applied in Embase).

PubMed / MEDLINE (NCBI PubMed)
Block 1 — Diclofenac
("Diclofenac"[Mesh] OR diclofenac[tiab] OR diclophenac[tiab] OR voltaren[tiab] OR voltarol[tiab] OR pennsaid[tiab] OR flector[tiab] OR licart[tiab] OR solaraze[tiab] OR diclac[tiab] OR cambia[tiab] OR cataflam[tiab] OR zipsor[tiab] OR zorvolex[tiab] OR olfen[tiab] OR algesal[tiab] OR dyloject[tiab] OR diclofen[tiab] OR dolex[tiab] OR naclof[tiab])
Block 2 — Topical administration
("Administration, Topical"[Mesh] OR "Administration, Cutaneous"[Mesh] OR "Skin Absorption"[Mesh] OR "Transdermal Patch"[Mesh] OR "Ointments"[Mesh] OR "Gels"[Mesh] OR topical*[tiab] OR transdermal*[tiab] OR transcutaneous[tiab] OR percutaneous*[tiab] OR cutaneous*[tiab] OR dermal*[tiab] OR epicutaneous[tiab] OR "skin application"[tiab] OR "skin absorption"[tiab] OR "skin penetration"[tiab] OR "local application"[tiab] OR "local treatment"[tiab] OR "local therapy"[tiab] OR "drug delivery system"[tiab] OR gel[tiab] OR gels[tiab] OR emulgel[tiab] OR cream[tiab] OR creams[tiab] OR ointment[tiab] OR ointments[tiab] OR lotion[tiab] OR lotions[tiab] OR solution[tiab] OR solutions[tiab] OR spray[tiab] OR sprays[tiab] OR patch[tiab] OR patches[tiab] OR plaster[tiab] OR plasters[tiab] OR foam[tiab] OR emulsion[tiab] OR liniment[tiab] OR aerosol[tiab] OR film-forming[tiab] OR iontophor*[tiab] OR phonophor*[tiab] OR sonophor*[tiab])
Block 3 — Renal outcome
("Acute Kidney Injury"[Mesh] OR "Renal Insufficiency"[Mesh] OR "Renal Insufficiency, Chronic"[Mesh] OR "Kidney Diseases"[Mesh] OR "Kidney Failure, Chronic"[Mesh] OR "Glomerular Filtration Rate"[Mesh] OR "Creatinine"[Mesh] OR "Urea"[Mesh] OR "Blood Urea Nitrogen"[Mesh] OR "Hyperkalemia"[Mesh] OR "Hypokalemia"[Mesh] OR "Water-Electrolyte Imbalance"[Mesh] OR "Edema"[Mesh] OR "Urinalysis"[Mesh] OR "Proteinuria"[Mesh] OR "Albuminuria"[Mesh] OR "Hematuria"[Mesh] OR "Lipocalin-2"[Mesh] OR "Hepatitis A Virus Cellular Receptor 1"[Mesh] OR "Cystatin C"[Mesh] OR "Renal Replacement Therapy"[Mesh] OR "Renal Dialysis"[Mesh] OR "Biopsy"[Mesh] OR "Nephritis,

Interstitial"[Mesh] OR "Kidney Tubular Necrosis, Acute"[Mesh] OR "kidney"[tiab] OR "kidneys"[tiab] OR "renal"[tiab] OR "renally"[tiab] OR "nephrotoxic"[tiab] OR "nephrotoxicity"[tiab] OR "nephrotoxin"[tiab] OR "nephrotoxins"[tiab] OR "nephritis"[tiab] OR "nephropathy"[tiab] OR "nephropathic"[tiab] OR "nephrosis"[tiab] OR "nephrotic"[tiab] OR "creatinine"[tiab] OR "serum creatinine"[tiab] OR "plasma creatinine"[tiab] OR "SCr"[tiab] OR "creatinine clearance"[tiab] OR "CrCl"[tiab] OR "glomerular filtration"[tiab] OR "eGFR"[tiab] OR "GFR"[tiab] OR "CKD-EPI"[tiab] OR "MDRD"[tiab] OR "Cockcroft-Gault"[tiab] OR "blood urea nitrogen"[tiab] OR "BUN"[tiab] OR "urea"[tiab] OR "serum urea"[tiab] OR "uremia"[tiab] OR "uraemia"[tiab] OR "azotemia"[tiab] OR "azotaemia"[tiab] OR "hyperkalemia"[tiab] OR "hyperkalaemia"[tiab] OR "hypokalemia"[tiab] OR "hypokalaemia"[tiab] OR "hyponatremia"[tiab] OR "hyponatraemia"[tiab] OR "hypernatremia"[tiab] OR "hypernatraemia"[tiab] OR "electrolyte imbalance"[tiab] OR "electrolyte disturbance"[tiab] OR "metabolic acidosis"[tiab] OR "tubular acidosis"[tiab] OR "Fanconi syndrome"[tiab] OR "uric acid"[tiab] OR "hyperuricemia"[tiab] OR "hyperuricaemia"[tiab] OR "fluid retention"[tiab] OR "edema"[tiab] OR "oedema"[tiab] OR "renal failure"[tiab] OR "kidney failure"[tiab] OR "acute renal failure"[tiab] OR "ARF"[tiab] OR "acute kidney injury"[tiab] OR "AKI"[tiab] OR "chronic kidney disease"[tiab] OR "CKD"[tiab] OR "chronic renal failure"[tiab] OR "CRF"[tiab] OR "renal insufficiency"[tiab] OR "kidney insufficiency"[tiab] OR "renal impairment"[tiab] OR "kidney impairment"[tiab] OR "renal disease"[tiab] OR "kidney disease"[tiab] OR "renal toxicity"[tiab] OR "kidney toxicity"[tiab] OR "renal adverse"[tiab] OR "kidney adverse"[tiab] OR "renal safety"[tiab] OR "kidney safety"[tiab] OR "dialysis"[tiab] OR "hemodialysis"[tiab] OR "haemodialysis"[tiab] OR "renal replacement therapy"[tiab] OR "RRT"[tiab] OR "KDIGO"[tiab] OR "AKIN"[tiab] OR "RIFLE"[tiab] OR "urinalysis"[tiab] OR "urine analysis"[tiab] OR "urinary sediment"[tiab] OR "urine sediment"[tiab] OR "urinary microscopy"[tiab] OR "dipstick"[tiab] OR "urine dipstick"[tiab] OR "urinary cast"[tiab] OR "urinary casts"[tiab] OR "granular cast"[tiab] OR "hyaline cast"[tiab] OR "leukocyte cast"[tiab] OR "erythrocyte cast"[tiab] OR "proteinuria"[tiab] OR "albuminuria"[tiab] OR "microalbuminuria"[tiab] OR "macroalbuminuria"[tiab] OR "albumin to creatinine ratio"[tiab] OR "ACR"[tiab] OR "albumin/creatinine"[tiab] OR "hematuria"[tiab] OR "haematuria"[tiab] OR "leucocyturia"[tiab] OR "leukocyturia"[tiab] OR "pyuria"[tiab] OR "eosinophiluria"[tiab] OR "glycosuria"[tiab] OR "aminoaciduria"[tiab] OR "phosphaturia"[tiab] OR "specific gravity"[tiab] OR "urinary osmolality"[tiab] OR "urine osmolality"[tiab] OR "urinary pH"[tiab] OR "urine pH"[tiab] OR "NGAL"[tiab] OR "neutrophil gelatinase"[tiab] OR "lipocalin-2"[tiab] OR "lipocalin 2"[tiab] OR "LCN2"[tiab] OR "KIM-1"[tiab] OR "kidney injury molecule"[tiab] OR "HAVCR1"[tiab] OR "L-FABP"[tiab] OR "liver-type fatty acid-binding"[tiab] OR "FABP1"[tiab] OR "interleukin-18"[tiab] OR "IL-18"[tiab] OR "beta-2 microglobulin"[tiab] OR "beta2-microglobulin"[tiab] OR "alpha-1 microglobulin"[tiab] OR "N-acetyl-beta-D-glucosaminidase"[tiab] OR "NAG"[tiab] OR "TIMP-2"[tiab] OR "IGFBP-7"[tiab] OR "cystatin C"[tiab] OR "cystatin-C"[tiab] OR "renal biopsy"[tiab] OR "kidney biopsy"[tiab] OR "interstitial nephritis"[tiab] OR "tubular necrosis"[tiab] OR "acute interstitial nephritis"[tiab] OR "ATN"[tiab] OR "papillary necrosis"[tiab] OR "analgesic nephropathy"[tiab] OR "Banff classification"[tiab] OR "renal ultrasound"[tiab] OR "kidney ultrasound"[tiab] OR "resistive index"[tiab] OR "renal CT"[tiab] OR "renal MRI"[tiab] OR "BOLD-MRI"[tiab] OR "arterial spin labelling"[tiab] OR "arterial spin labeling"[tiab])

Web of Science Core Collection (Clarivate)

Block 1 — Diclofenac

TS=(diclofenac OR diclophenac OR voltaren OR voltarol OR pennsaid OR flector OR licart OR solaraze OR diclac OR cambia OR cataflam OR zipsor OR zorvolex OR dicloreum OR dicloflex OR difene OR modifenac OR vostar OR olfen OR algesal OR dyloject OR diclofen OR dolex OR diclonat OR naclof)

Block 2 — Topical administration

TS=(topical* OR transdermal* OR transcutaneous OR percutaneous* OR cutaneous* OR dermal* OR epicutaneous OR "skin application" OR "skin absorption" OR "skin penetration" OR "local application" OR

"local treatment" OR "local therapy" OR "drug delivery system" OR gel OR gels OR emulgel OR cream OR creams OR ointment OR ointments OR lotion OR lotions OR solution OR solutions OR spray OR sprays OR patch OR patches OR plaster OR plasters OR foam OR emulsion OR liniment OR aerosol OR "film-forming" OR iontophor* OR phonophor* OR sonophor*)

Block 3 — Renal outcome

TS=(kidney OR kidneys OR renal OR renally OR nephrotoxic OR nephrotoxicity OR nephrotoxin OR nephrotoxins OR nephritis OR nephropathy OR nephropathic OR nephrosis OR nephrotic OR creatinine OR "serum creatinine" OR "plasma creatinine" OR SCr OR "creatinine clearance" OR CrCl OR "glomerular filtration" OR eGFR OR GFR OR "CKD-EPI" OR MDRD OR "Cockcroft-Gault" OR "blood urea nitrogen" OR BUN OR urea OR "serum urea" OR uremia OR uraemia OR azotemia OR azotaemia OR hyperkalemia OR hyperkalaemia OR hypokalemia OR hypokalaemia OR hyponatremia OR hyponatraemia OR hypernatremia OR hypernatraemia OR "electrolyte imbalance" OR "electrolyte disturbance" OR "metabolic acidosis" OR "tubular acidosis" OR "Fanconi syndrome" OR "uric acid" OR hyperuricemia OR hyperuricaemia OR "fluid retention" OR edema OR oedema OR "renal failure" OR "kidney failure" OR "acute renal failure" OR ARF OR "acute kidney injury" OR AKI OR "chronic kidney disease" OR CKD OR "chronic renal failure" OR CRF OR "renal insufficiency" OR "kidney insufficiency" OR "renal impairment" OR "kidney impairment" OR "renal disease" OR "kidney disease" OR "renal toxicity" OR "kidney toxicity" OR "renal adverse" OR "kidney adverse" OR "renal safety" OR "kidney safety" OR dialysis OR hemodialysis OR haemodialysis OR "renal replacement therapy" OR RRT OR KDIGO OR AKIN OR RIFLE OR urinalysis OR "urine analysis" OR "urinary sediment" OR "urine sediment" OR "urinary microscopy" OR dipstick OR "urine dipstick" OR "urinary cast" OR "urinary casts" OR "granular cast" OR "hyaline cast" OR "leukocyte cast" OR "erythrocyte cast" OR proteinuria OR albuminuria OR microalbuminuria OR macroalbuminuria OR "albumin to creatinine ratio" OR ACR OR hematuria OR haematuria OR leucocyturia OR leukocyturia OR pyuria OR eosinophiluria OR glycosuria OR aminoaciduria OR phosphaturia OR "specific gravity" OR "urinary osmolality" OR "urine osmolality" OR "urinary pH" OR "urine pH" OR NGAL OR "neutrophil gelatinase" OR "lipocalin-2" OR "lipocalin 2" OR LCN2 OR "KIM-1" OR "kidney injury molecule" OR HAVCR1 OR "L-FABP" OR "liver-type fatty acid-binding" OR FABP1 OR "interleukin-18" OR "IL-18" OR "beta-2 microglobulin" OR "beta2-microglobulin" OR "alpha-1 microglobulin" OR "N-acetyl-beta-D-glucosaminidase" OR NAG OR "TIMP-2" OR "IGFBP-7" OR "cystatin C" OR "cystatin-C" OR "renal biopsy" OR "kidney biopsy" OR "interstitial nephritis" OR "tubular necrosis" OR "acute interstitial nephritis" OR ATN OR "papillary necrosis" OR "analgesic nephropathy" OR "Banff classification" OR "renal ultrasound" OR "kidney ultrasound" OR "resistive index" OR "renal CT" OR "renal MRI" OR "BOLD-MRI" OR "arterial spin labelling" OR "arterial spin labeling")

Scopus (Elsevier)

Block 1 — Diclofenac

TITLE-ABS-KEY(diclofenac OR diclophenac OR voltaren OR voltarol OR pennsaid OR flector OR licart OR solaraze OR diclac OR cambia OR cataflam OR zipsor OR zorvolex OR dicloreum OR dicloflex OR difene OR modifenac OR vostar OR olfen OR algesal OR dyloject OR diclofen OR dolex OR diclonat OR naclof)

Block 2 — Topical administration

TITLE-ABS-KEY(topical* OR transdermal* OR transcutaneous OR percutaneous* OR cutaneous* OR dermal* OR epicutaneous OR "skin application" OR "skin absorption" OR "skin penetration" OR "local application" OR "local treatment" OR "local therapy" OR "drug delivery system" OR gel OR gels OR emulgel OR cream OR creams OR ointment OR ointments OR lotion OR lotions OR solution OR solutions OR spray OR sprays OR patch OR patches OR plaster OR plasters OR foam OR emulsion OR liniment OR aerosol OR "film-forming" OR iontophor* OR phonophor* OR sonophor*)

Block 3 — Renal outcome

TITLE-ABS-KEY(kidney OR kidneys OR renal OR renally OR nephrotoxic OR nephrotoxicity OR nephrotoxin OR nephrotoxins OR nephritis OR nephropathy OR nephropathic OR nephrosis OR nephrotic OR creatinine OR "serum creatinine" OR "plasma creatinine" OR SCr OR "creatinine clearance" OR CrCl OR "glomerular filtration" OR eGFR OR GFR OR "CKD-EPI" OR MDRD OR "Cockcroft-Gault" OR "blood urea nitrogen" OR BUN OR urea OR "serum urea" OR uremia OR uraemia OR azotemia OR azotaemia OR hyperkalemia OR hyperkalaemia OR hypokalemia OR hypokalaemia OR hyponatremia OR hyponatraemia OR hypernatremia OR hypernatraemia OR "electrolyte imbalance" OR "electrolyte disturbance" OR "metabolic acidosis" OR "tubular acidosis" OR "Fanconi syndrome" OR "uric acid" OR hyperuricemia OR hyperuricaemia OR "fluid retention" OR edema OR oedema OR "renal failure" OR "kidney failure" OR "acute renal failure" OR ARF OR "acute kidney injury" OR AKI OR "chronic kidney disease" OR CKD OR "chronic renal failure" OR CRF OR "renal insufficiency" OR "kidney insufficiency" OR "renal impairment" OR "kidney impairment" OR "renal disease" OR "kidney disease" OR "renal toxicity" OR "kidney toxicity" OR "renal adverse" OR "kidney adverse" OR "renal safety" OR "kidney safety" OR dialysis OR hemodialysis OR haemodialysis OR "renal replacement therapy" OR RRT OR KDIGO OR AKIN OR RIFLE OR urinalysis OR "urine analysis" OR "urinary sediment" OR "urine sediment" OR "urinary microscopy" OR dipstick OR "urine dipstick" OR "urinary cast" OR "urinary casts" OR "granular cast" OR "hyaline cast" OR "leukocyte cast" OR "erythrocyte cast" OR proteinuria OR albuminuria OR microalbuminuria OR macroalbuminuria OR "albumin to creatinine ratio" OR ACR OR hematuria OR haematuria OR leucocyturia OR leukocyturia OR pyuria OR eosinophiluria OR glycosuria OR aminoaciduria OR phosphaturia OR "specific gravity" OR "urinary osmolality" OR "urine osmolality" OR "urinary pH" OR "urine pH" OR NGAL OR "neutrophil gelatinase" OR "lipocalin-2" OR "lipocalin 2" OR LCN2 OR "KIM-1" OR "kidney injury molecule" OR HAVCR1 OR "L-FABP" OR "liver-type fatty acid-binding" OR FABP1 OR "interleukin-18" OR "IL-18" OR "beta-2 microglobulin" OR "beta2-microglobulin" OR "alpha-1 microglobulin" OR "N-acetyl-beta-D-glucosaminidase" OR NAG OR "TIMP-2" OR "IGFBP-7" OR "cystatin C" OR "cystatin-C" OR "renal biopsy" OR "kidney biopsy" OR "interstitial nephritis" OR "tubular necrosis" OR "acute interstitial nephritis" OR ATN OR "papillary necrosis" OR "analgesic nephropathy" OR "Banff classification" OR "renal ultrasound" OR "kidney ultrasound" OR "resistive index" OR "renal CT" OR "renal MRI" OR "BOLD-MRI" OR "arterial spin labelling" OR "arterial spin labeling")

Embase (Embase.com)

Block 1 — Diclofenac

'diclofenac'/exp OR (diclofenac OR diclophenac OR voltaren OR voltarol OR pennsaid OR flector OR licart OR solaraze OR diclac OR cambia OR cataflam OR zipsor OR zorvolex OR dicloreum OR dicloflex OR difene OR modifenac OR vostar OR olfen OR algesal OR dyloject OR diclofen OR dolex OR diclonat OR naclof):ti,ab,kw

Block 2 — Topical administration

('topical drug administration'/exp OR 'transdermal drug administration'/exp OR 'percutaneous drug administration'/exp OR 'skin penetration'/exp OR 'transdermal patch'/exp OR 'gel'/exp OR 'cream'/exp OR 'ointment'/exp) OR (topical* OR transdermal* OR transcutaneous OR percutaneous* OR cutaneous* OR dermal* OR epicutaneous OR 'skin application' OR 'skin absorption' OR 'skin penetration' OR 'local application' OR 'local treatment' OR 'local therapy' OR 'drug delivery system' OR gel OR gels OR emulgel OR cream OR creams OR ointment OR ointments OR lotion OR lotions OR solution OR solutions OR spray OR sprays OR patch OR patches OR plaster OR plasters OR foam OR emulsion OR liniment OR aerosol OR 'film-forming' OR iontophor* OR phonophor* OR sonophor*):ti,ab,kw

Block 3 — Renal outcome

('acute kidney failure'/exp OR 'kidney failure'/exp OR 'chronic kidney failure'/exp OR 'kidney disease'/exp OR 'kidney function'/exp OR 'nephrotoxicity'/exp OR 'creatinine'/exp OR 'creatinine blood level'/exp OR 'glomerulus filtration rate'/exp OR 'urea'/exp OR 'urea nitrogen blood level'/exp OR 'hyperkalemia'/exp OR

'hypokalemia'/exp OR 'hyponatremia'/exp OR 'electrolyte disturbance'/exp OR 'edema'/exp OR 'urinalysis'/exp OR 'proteinuria'/exp OR 'hematuria'/exp OR 'albuminuria'/exp OR 'cystatin C'/exp OR 'neutrophil gelatinase associated lipocalin'/exp OR 'kidney injury molecule 1'/exp OR 'kidney biopsy'/exp OR 'hemodialysis'/exp OR 'kidney replacement therapy'/exp OR 'interstitial nephritis'/exp OR 'kidney tubule necrosis'/exp) OR (kidney OR kidneys OR renal OR renally OR nephrotoxic OR nephrotoxicity OR nephrotoxin OR nephrotoxins OR nephritis OR nephropathy OR nephropathic OR nephrosis OR nephrotic OR creatinine OR 'serum creatinine' OR 'plasma creatinine' OR SCr OR 'creatinine clearance' OR CrCl OR 'glomerular filtration' OR eGFR OR GFR OR 'CKD-EPI' OR MDRD OR 'Cockcroft-Gault' OR 'blood urea nitrogen' OR BUN OR urea OR 'serum urea' OR uremia OR uraemia OR azotemia OR azotaemia OR hyperkalemia OR hyperkalaemia OR hypokalemia OR hypokalaemia OR hyponatremia OR hyponatraemia OR hypernatremia OR hypernatraemia OR 'electrolyte imbalance' OR 'electrolyte disturbance' OR 'metabolic acidosis' OR 'tubular acidosis' OR 'Fanconi syndrome' OR 'uric acid' OR hyperuricemia OR hyperuricaemia OR 'fluid retention' OR edema OR oedema OR 'renal failure' OR 'kidney failure' OR 'acute renal failure' OR ARF OR 'acute kidney injury' OR AKI OR 'chronic kidney disease' OR CKD OR 'chronic renal failure' OR CRF OR 'renal insufficiency' OR 'kidney insufficiency' OR 'renal impairment' OR 'kidney impairment' OR 'renal disease' OR 'kidney disease' OR 'renal toxicity' OR 'kidney toxicity' OR 'renal adverse' OR 'kidney adverse' OR 'renal safety' OR 'kidney safety' OR dialysis OR hemodialysis OR haemodialysis OR 'renal replacement therapy' OR RRT OR KDIGO OR AKIN OR RIFLE OR urinalysis OR 'urine analysis' OR 'urinary sediment' OR 'urine sediment' OR 'urinary microscopy' OR dipstick OR 'urine dipstick' OR proteinuria OR albuminuria OR microalbuminuria OR macroalbuminuria OR 'albumin to creatinine ratio' OR ACR OR hematuria OR haematuria OR leucocyturia OR leukocyturia OR pyuria OR eosinophiluria OR glycosuria OR aminoaciduria OR phosphaturia OR 'urinary osmolality' OR 'urine osmolality' OR 'urinary pH' OR 'urine pH' OR NGAL OR 'neutrophil gelatinase' OR 'lipocalin-2' OR LCN2 OR 'KIM-1' OR 'kidney injury molecule' OR HAVCR1 OR 'L-FABP' OR FABP1 OR 'interleukin-18' OR 'IL-18' OR 'beta-2 microglobulin' OR 'alpha-1 microglobulin' OR 'N-acetyl-beta-D-glucosaminidase' OR NAG OR 'TIMP-2' OR 'IGFBP-7' OR 'cystatin C' OR 'cystatin-C' OR 'renal biopsy' OR 'kidney biopsy' OR 'interstitial nephritis' OR 'tubular necrosis' OR 'acute interstitial nephritis' OR ATN OR 'papillary necrosis' OR 'analgesic nephropathy' OR 'renal ultrasound' OR 'kidney ultrasound' OR 'resistive index'):ti,ab,kw

Non-human exclusion (final step)

NOT ('animal'/exp NOT 'human'/exp)

S1.3. Records identified and selection results

Records identified per database

**Database / source**	**Records identified (n)**
PubMed/MEDLINE	457
Scopus	2098
Embase	1899
Web of Science	671
**Total identified**	**5125**

Screening and selection stages

**Stage**	**n**
Records identified (all databases)	5125
Duplicate records removed	1154

Non-human records removed	1549
Records screened (title and abstract)	2422
Records excluded at title and abstract	2329
Reports assessed for eligibility (full text)	93
Excluded at full text — wrong intervention	15
Excluded at full text — wrong outcome	49
Excluded at full text — wrong population	11
**Studies included in the review**	**18**

Two reports excluded at full text are noted because their exposure and design were otherwise eligible and only the absence of a renal outcome excluded them: a phase I pharmacokinetic study limited to urinary diclofenac recovery (Andersen 2025) and a placebo-controlled epolamine-patch trial whose only renal mention was an exclusion criterion (Brühlmann 2003); both fall within the wrong-outcome category of the flow diagram.

S1.4. Search peer review

Each database-specific search strategy was internally checked against the PRESS 2015 guideline, including controlled-vocabulary mapping, Boolean logic, syntax, translation across databases, and application of limits. Formal external PRESS peer review by an information specialist was not performed.
