# Supplementary material for: Renal Safety of Topical Diclofenac in Adults with Increased Renal Risk: A Systematic Review
Source: Medicina (Kaunas). 2026 Aug 21;62(8):1606. doi: 10.3390/medicina62081606 (PMC13515200; doi:10.3390/medicina62081606)
Supplement: Supplementary file 1 [file medicina-62-01606-s001.zip › Supplementary Material S2.pdf]

**Supplementary Material S2**

*Complete renal dataset*

This file supports the systematic review "Renal Safety of Topical Diclofenac in Adults with Increased Renal Risk: A Systematic Review." It reports every renal observation extracted from the included studies, including subgroup figures, by-arm shifts, and derived values, with the source study indicated for each row.

**S2.1. Extracted renal observations**

**Table S1.** Complete renal dataset extracted from the included studies. AE, adverse event; AKI, acute kidney injury; CrCl, creatinine clearance; CV, cardiovascular; DBP, diastolic blood pressure; eGFR, estimated glomerular filtration rate; NS, not significant; SBP, systolic blood pressure; TXB2, thromboxane B2. Derived values are calculated from reported group means (1 mg/dL = 88.4 μmol/L). Empty study cells continue the study named in the row above.

| Study | Outcome | Topical value | Comparator / subgroup detail |
|---|---|---|---|
| **Teo 2023 [72]** | AKI any (KDIGO) | adj OR 1.38 (1.18–1.63); 18.9% | No-NSAID 14.5%; systemic adj OR 1.77 (1.46–2.15), 19.2%; p<0.001 |
| | AKI stage 2/3 | adj OR 1.29 (0.89–1.88), NS | Systemic adj OR 1.68 (1.09–2.58), p=0.02 |
| | Hyperkalaemia | 8.6%; adj OR 1.03 (0.83–1.28), NS | No-NSAID 8.0%; systemic 8.3%, adj OR 1.15 (0.88–1.50), NS; p=0.73 |
| | Nephrology consult | 12.8%; adj OR 1.69 (1.39–2.10), p<0.001 | No-NSAID 9.4%; systemic 10.0%, adj OR 1.41–1.43 |
| | Other AKI predictors (model) | — | Diuretic adj OR 2.63; recent CV hospitalisation 1.80; lower baseline eGFR (per mL/min 0.98) |
| | Baseline eGFR | 41.9 mL/min/1.73 m² (G3 79%, G4 21%) | Δ eGFR over follow-up not reported |
| **Sicignano 2025 [73]** | Renal composite (ICD) | 3.3% (17,158/521,593) | Renal-risk subgroup 7.7%; diabetes 7.5%; age ≥85 8.6%; 75–84 7.1%; 65–74 4.9% |
| | Concomitant oral NSAID | 55.4% of renal-event cohort | — |
| | Incident hypertension | 4.5% | Fluid retention/oedema not separately reported |
| | Healthcare use (renal-event) | 37–55 outpatient, 3.2–7.4 ED visits | ICD-coded, not adjudicated |
| **Roth 2012 (≥75 y) [74]** | Δ serum creatinine | ≈+0.5 μmol/L (NS) | Placebo ≈+2.6; control ≈+3.8; all <4.4 μmol/L (<0.05 mg/dL) |
| | Renal/urinary AE | 0/138 (0.0%) | Placebo 0/39 (0.0%); control 2/103 (1.9%); NS |
| | Blood pressure (Δ) | SBP −3.61, DBP −1.13 | Placebo SBP −0.32, DBP −3.11; all NS |
| **Baraf 2012 [76]** | Renal/CV AE (subgroups) | No excess in older/HTN/diabetic/CV subgroups | Worsening hypertension ~1–1.3% (knee gel); oedema not separately reported |
| | Subgroup sizes (knee) | ≥65 y 274 DSG; HTN; T2DM 193; CVD 67 | Vehicle ≥65 y 264 |
| **Peniston 2012 [65]** | Peripheral oedema (subgroups) | T2DM 5.0%; CVD 3.1%; multicomorbidity 6.7% | Without T2DM 1.1%; without CVD 1.3%; HTN 1.4% vs 1.6% |

| | | | |
|---|---|---|---|
| | CV adverse events | HTN 5.0%; T2DM 8.0%; CVD 6.2%; multicomorbidity 13.3% | Without comorbidity ~1.8–3.1% |
| | Renal laboratory values | Not reported (collected, unreported) | Subgroups: ≥65 n=372, HTN 438, T2DM 100, CVD 97 |
| **Tugwell 2004 [51]** | Δ serum creatinine | +0.3 (11) µmol/L | Oral +3.3 (13); p=0.003 (ANOVA) |
| | Δ creatinine clearance | +0.6 (16) mL/min | Oral −2.7 (12); p=0.006 |
| | Abnormal CrCl shift | 12/281 (4%) | Oral 27/277 (10%); p=0.01 |
| | Oedema / hypertension AE | oedema 7%; HTN 1% | Oral oedema 8% (p=0.65); HTN 2% (p=0.20) |
| **Simon 2009 [52]** | Δ serum creatinine | +0.4 (10.5) µmol/L | Placebo +0.8; DMSO +0.3; oral +3.1; combination +4.4 (11.2) |
| | Abnormal creatinine shift | 2.8% (4/145) | Placebo 5.6%; DMSO 4.0%; oral 7.2%; combination 10.6% |
| | Δ creatinine clearance | +0.4 mL/min | Placebo +0.5; DMSO +0.5; oral +2.4; combination +3.3 |
| | Abnormal CrCl shift | 11/145 (7.6%) | Placebo 5.7%; DMSO 6.0%; oral 7.2%; combination 11.4% |
| | Incident hypertension AE | 1.3% | Placebo 0.6%; CV events <2%/arm |
| **Roth 2011 (JPR) [53]** | Abnormal creatinine shift | 8.2% | Oral 10.3%; p=0.30 (NS) despite significant mean difference |
| | Liver/renal-test discontinuation | 1 (0.2%) | Oral 5 (1.1%); renal not isolated |
| | Diastolic BP ≥90 mmHg | 34.9% | Oral 44.3%; p<0.01 |
| **Roth 2011 (PGM) [75]** | Δ serum creatinine | ≈+0.4 µmol/L (negligible) | Placebo ≈−0.1; NS |
| | Δ creatinine clearance | +0.6 mL/min | Placebo 0.0; comparable |
| | Blood pressure (Δ) | SBP −1.8, DBP −1.1 | Placebo SBP −2.4, DBP −1.7; no HTN signal |
| **Roth 2004 [66]** | Renal AE composite | 0 (explicit, both arms) | Vehicle 0; 'no related renal AE in any group' |
| | Oedema | 4 (2.4%) | Vehicle 2 (1.2%); NS |
| **Bookman 2004 [63]** | Renal AE (narrative) | None serious; no lab-detected renal AE | Urine sampled, not reported numerically |
| **Altman 2009 [62]** | Renal events (narrative) | Severe renal events seen with oral 'did not occur' | No numeric counts; no BP change |
| **Niethard 2005 [64]** | Urinary measures (narrative) | 'No changes of note' | Urine sampled, not reported |
| **Baheti 2025 [68]** | Δ serum creatinine (within-arm) | ≈+10.6 µmol/L (derived, perioperative) | Ketoprofen patch comparator; no AKI. Rise occurs after percutaneous nephrolithotomy and is most plausibly surgical, not diclofenac-attributable. |

| | | | |
|---|---|---|---|
| **Ahmadabadi 2020 [71]** | Δ serum creatinine (within-arm) | +1.63 µmol/L, p=0.047 (within-arm) | Between-arm p=0.194 (NS); BUN measured |
| **Peniston 2011 [70]** | Treatment-related renal AE | 1 patient ('creatinine elevated') | First treatment-attributed topical renal AE in the set |
| | Peripheral oedema | 1.9% (treatment-emergent) | Not attributed to study drug |
| **Hellms 2019 [69]** | Renal perfusion / eGFR | Topical eGFR 91→99 (NS); no TXB2 suppression | Oral eGFR 91→90 (NS); creatinine within normal range |
| **Kienzler 2010 [67]** | Renal AE composite | 0 (qualitative) | No renal monitoring performed; baseline normal |
