# Supplementary material for: Renal Safety of Topical Diclofenac in Adults with Increased Renal Risk: A Systematic Review
Source: Medicina (Kaunas). 2026 Aug 21;62(8):1606. doi: 10.3390/medicina62081606 (PMC13515200; doi:10.3390/medicina62081606)
Supplement: Supplementary file 1 [file medicina-62-01606-s001.zip › Supplementary Material S3. PRISMA_2020_checklist_Renal_Safety_Topical_Diclofenac.pdf]

PRISMA

PRISMA 2020 Checklist

Section and Topic	Item #	Checklist item	Location where item is reported
**TITLE**			
Title	1	Identify the report as a systematic review.	Title (p. 1)
**ABSTRACT**			
Abstract	2	See the PRISMA 2020 for Abstracts checklist.	Abstract (p. 1): Background and Objectives; Materials and Methods; Results; Conclusions
**INTRODUCTION**			
Rationale	3	Describe the rationale for the review in the context of existing knowledge.	Section 1.1 (Rationale); Section 1.2 (Knowledge Gap)
Objectives	4	Provide an explicit statement of the objective(s) or question(s) the review addresses.	Section 1.3 (Objectives); Section 1.4 (PECO Framework)
**METHODS**			
Eligibility criteria	5	Specify the inclusion and exclusion criteria for the review and how studies were grouped for the syntheses.	Section 2.2 (Eligibility Criteria), including 2.2.1 (Inclusion criteria) and 2.2.2 (Exclusion criteria); grouping into the two predefined strata (increased renal risk; general or healthy-volunteer) in Sections 2.2 and 2.5
Information sources	6	Specify all databases, registers, websites, organisations, reference lists and other sources searched or consulted to identify studies. Specify the date when each source was last searched or consulted.	Section 2.3 (Information Sources): databases, trial registries and pharmacovigilance sources, citation chasing and reference screening; search period (inception to 19 April 2026) in Section 2.1; platforms and execution date in Supplementary Material S1, Section S1.1
Search strategy	7	Present the full search strategies for all databases, registers and websites, including any filters and limits used.	Section 2.4 (Search Strategy); full database-specific strategies, reproduced as entered with all blocks, filters and limits, in Supplementary Material S1, Section S1.2; internal PRESS 2015 check in Section S1.4
Selection process	8	Specify the methods used to decide whether a study met the inclusion criteria of the review, including how many reviewers screened each record and each report retrieved, whether they worked independently, and if applicable, details of automation tools used in the process.	Section 2.5 (Study Selection, Extraction and Synthesis of Data): two reviewers screened titles, abstracts and full texts independently, with third-reviewer adjudication; no automated screening or text-mining tools were used
Data collection process	9	Specify the methods used to collect data from reports, including how many reviewers collected data from each report, whether they worked independently, any processes for obtaining or confirming data from study investigators, and if applicable, details of automation tools used in the process.	Section 2.5: duplicate independent extraction using a piloted standardised form, consensus resolution, and a defined procedure for contacting study authors; no automated data-extraction tools were used
Data items	10a	List and define all outcomes for which data were sought. Specify whether all results that were compatible with each outcome domain in each study were sought (e.g. for all measures, time points, analyses), and if not, the methods used to decide which results to collect.	Section 1.4 (PECO; renal outcomes organised into four predefined tiers); Section 2.2 (requirement for at least one reported renal outcome); Section 2.5 (which results were collected for each outcome); outcomes reported per study in Section 3.6 and in Supplementary Material S2, Table S1
	10b	List and define all other variables for which data were sought (e.g. participant and intervention characteristics, funding sources). Describe any assumptions made about any missing or unclear information.	Sections 1.4 and 2.2 (population, exposure and comparator variables); Section 3.3 and Table 4 (design, population, formulation, comparator and renal data extracted for each study); provenance and sponsorship of the included studies in Section 4.7
Study risk of	11	Specify the methods used to assess risk of bias in the included studies, including details of the tool(s)	Section 2.6 (Risk-of-bias and Certainty Assessment): RoB 2 including the crossover extension, ROBINS-I,

Section and Topic	Item #	Checklist item	Location where item is reported
bias assessment		used, how many reviewers assessed each study and whether they worked independently, and if applicable, details of automation tools used in the process.	and the JBI critical appraisal checklist for case series; two independent reviewers with third-reviewer adjudication; no automation tools were used
Effect measures	12	Specify for each outcome the effect measure(s) (e.g. risk ratio, mean difference) used in the synthesis or presentation of results.	Section 2.5: mean change from baseline with corresponding measures of dispersion for continuous outcomes; risk ratios or odds ratios with 95% confidence intervals for dichotomous outcomes, extracted as reported
Synthesis methods	13a	Describe the processes used to decide which studies were eligible for each synthesis (e.g. tabulating the study intervention characteristics and comparing against the planned groups for each synthesis (item #5)).	Sections 2.2 and 2.5 (assignment of studies to the two predefined strata and to each synthesis); implemented in Section 3.3 and Table 4
	13b	Describe any methods required to prepare the data for presentation or synthesis, such as handling of missing summary statistics, or data conversions.	Section 2.5 (no statistical transformation for pooling; absolute values derived from published event counts, see also Section 3.6); derived values identified in the legend of Table S1, Supplementary Material S2
	13c	Describe any methods used to tabulate or visually display results of individual studies and syntheses.	Section 2.5 (structured summary tables and a non-pooled forest plot); implemented in Tables 1-6 and Figures 2-9
	13d	Describe any methods used to synthesize results and provide a rationale for the choice(s). If meta-analysis was performed, describe the model(s), method(s) to identify the presence and extent of statistical heterogeneity, and software package(s) used.	Section 2.5: synthesis without meta-analysis (SWiM), direction-of-effect synthesis with a prespecified three-dataset threshold, and an itemised rationale for not pooling
	13e	Describe any methods used to explore possible causes of heterogeneity among study results (e.g. subgroup analysis, meta-regression).	Section 2.5 (a priori stratification by baseline renal risk; concordance versus divergence between strata interpreted as effect modification); results in Section 3.8
	13f	Describe any sensitivity analyses conducted to assess robustness of the synthesized results.	Section 2.6 (prespecified sensitivity analysis restricted to studies at low or moderate renal-outcome risk of bias)
Reporting bias assessment	14	Describe any methods used to assess risk of bias due to missing results in a synthesis (arising from reporting biases).	Section 2.6: risk of bias due to missing evidence assessed narratively with the ROB-ME framework, applied per synthesised outcome; funnel-plot and regression-based tests not applicable in the absence of pooling
Certainty assessment	15	Describe any methods used to assess certainty (or confidence) in the body of evidence for an outcome.	Section 2.6: GRADE applied independently by two reviewers, separately for each population stratum and each main renal outcome, with a ROBINS-I-based starting point
**RESULTS**			
Study selection	16a	Describe the results of the search and selection process, from the number of records identified in the search to the number of studies included in the review, ideally using a flow diagram.	Section 3.1 (Study Selection) and Figure 1 (PRISMA 2020 flow diagram); stage-by-stage retrieval and selection counts in Supplementary Material S1, Section S1.3
	16b	Cite studies that might appear to meet the inclusion criteria, but which were excluded, and explain why they were excluded.	Section 3.1 and Figure 1 (reports excluded at full text, with reasons by category); borderline full-text exclusions identified individually in Supplementary Material S1, Section S1.3
Study characteristics	17	Cite each included study and present its characteristics.	Section 3.3 (Characteristics of Included Studies) and Table 4; complete extracted renal dataset in Supplementary Material S2, Table S1

PRISMA 2020 Checklist

Section and Topic	Item #	Checklist item	Location where item is reported
Risk of bias in studies	18	Present assessments of risk of bias for each included study.	Section 3.2 (Risk of Bias of Included Studies); Tables 1-3; Figures 2 and 3 (domain-level traffic-light plots)
Results of individual studies	19	For all outcomes, present, for each study: (a) summary statistics for each group (where appropriate) and (b) an effect estimate and its precision (e.g. confidence/credible interval), ideally using structured tables or plots.	Sections 3.4-3.6 and Figures 4-7; complete study-level renal dataset, including subgroup and by-arm values, in Supplementary Material S2, Table S1
Results of syntheses	20a	For each synthesis, briefly summarise the characteristics and risk of bias among contributing studies.	Sections 3.3 and 3.6 (characteristics of the contributing studies for each synthesis); risk of bias of the contributing studies in Section 3.2 and Table 1
	20b	Present results of all statistical syntheses conducted. If meta-analysis was done, present for each the summary estimate and its precision (e.g. confidence/credible interval) and measures of statistical heterogeneity. If comparing groups, describe the direction of the effect.	Sections 3.4-3.6: no meta-analysis was performed; SWiM synthesis, direction of effect, and derived absolute estimates are presented, with study-level data in Supplementary Material S2, Table S1
	20c	Present results of all investigations of possible causes of heterogeneity among study results.	Section 3.8 (Comparison Across Populations), Table 5 and Figures 8 and 9; overlapping populations addressed in Section 3.7 and Table 3
	20d	Present results of all sensitivity analyses conducted to assess the robustness of the synthesized results.	Section 3.2 (results of the prespecified sensitivity analysis restricted to studies at lower renal-outcome risk of bias)
Reporting biases	21	Present assessments of risk of bias due to missing results (arising from reporting biases) for each synthesis assessed.	Section 3.2 (ROB-ME assessment: renal parameters measured but reported only qualitatively); carried forward as a qualifier in Section 4.7
Certainty of evidence	22	Present assessments of certainty (or confidence) in the body of evidence for each outcome assessed.	Section 3.9 (Certainty of Evidence) and Table 6 (GRADE Summary of Findings, by stratum and outcome)
**DISCUSSION**			
Discussion	23a	Provide a general interpretation of the results in the context of other evidence.	Sections 4.1-4.4
	23b	Discuss any limitations of the evidence included in the review.	Sections 4.3 and 4.7 (Strengths and Limitations of the Evidence Base)
	23c	Discuss any limitations of the review processes used.	Section 4.6 (Limitations)
	23d	Discuss implications of the results for practice, policy, and future research.	Section 4.5 (Implications for Clinical Practice and Guidelines); Section 4.3 (design required to resolve the measurement gap); Section 5 (Conclusions)
**OTHER INFORMATION**			
Registration and protocol	24a	Provide registration information for the review, including register name and registration number, or state that the review was not registered.	Section 2.1: PROSPERO, registration number CRD420261393454
	24b	Indicate where the review protocol can be accessed, or state that a protocol was not prepared.	Section 2.1: the protocol is publicly accessible through the PROSPERO registry under the same registration number
	24c	Describe and explain any amendments to information provided at registration or in the protocol.	Section 2.1: no amendments were made to the registered protocol during the conduct of the review
Support	25	Describe sources of financial or non-financial support for the review, and the role of the funders or sponsors in the review.	Funding statement (back matter); Acknowledgments
Competing interests	26	Declare any competing interests of review authors.	Conflicts of Interest statement (back matter)

PRISMA 2020 Checklist

Section and Topic	Item #	Checklist item	Location where item is reported
Availability of data, code and other materials	27	Report which of the following are publicly available and where they can be found: template data collection forms; data extracted from included studies; data used for all analyses; analytic code; any other materials used in the review.	Data Availability Statement and Supplementary Materials statement (back matter); full search strategies and stage-by-stage retrieval counts in Supplementary Material S1; complete extracted renal dataset in Supplementary Material S2, Table S1

Note. Locations are given by the section, table and figure numbering of the revised manuscript (medicina-4425506) and of Supplementary Materials S1 and S2. Section-level referencing is used because pagination differs between the submitted file and the typeset proof, whereas the section, table and figure numbers are stable.

From: Page MJ, McKenzie JE, Bossuyt PM, Boutron I, Hoffmann TC, Mulrow CD, et al. The PRISMA 2020 statement: an updated guideline for reporting systematic reviews. BMJ 2021;372:n71. doi: 10.1136/bmj.n71. This work is licensed under CC BY 4.0. To view a copy of this license, visit https://creativecommons.org/licenses/by/4.0/
